# A *de novo* Mutation in the *MTUS1* Gene Decreases the Risk of Non-compaction of Ventricular Myocardium via the Rac1/Cdc42 Pathway

**DOI:** 10.3389/fped.2019.00247

**Published:** 2019-07-02

**Authors:** Xuehan Bai, Yuanlin Zhou, Na Ouyang, Lingjuan Liu, Xupei Huang, Jie Tian, Tiewei Lv

**Affiliations:** ^1^Department of Cardiology, Children's Hospital of Chongqing Medical University, Chongqing, China; ^2^China International Science and Technology Cooperation Base of Child Development and Critical Disorders, Chongqing Key Laboratory of Pediatrics, Ministry of Education Key Laboratory of Child Development and Disorders, Chongqing, China; ^3^Chengdu Women's and Children's Central Hospital, Chengdu, China; ^4^Department of Biomedical Science, Charlie E. Schmidt College of Medicine, Florida Atlantic University, Boca Raton, FL, United States

**Keywords:** non-compaction of ventricular myocardium, MTUS1, microtubule, cell polarity, Rac1/Cdc42

## Abstract

**Background:** The *MTUS1* gene encodes a microtubule-associated protein involved in multiple processes including cell polarity and microtubule balance during myocardial development.

**Aims:** To investigate the association between a *de novo* c. 2617A->C mutation in *MTUS1* (NM_001001924.2) and non-compaction of ventricular myocardium (NVM) and explore the potential mechanisms.

**Methods:** A *de novo* mutation in *MTUS1* was identified for a familial pedigree with NVM. Lentiviral vectors containing *MTUS1* wild type or the mutation *MTUS1* were constructed and co-infected into HEK-293 cells. *MTUS1*, Rac1/Cdc42, α-tubulin, α/β-tubulin, polarity protein (PAR6), and the morphology of daughter cells were measured by real-time PCR, Western blot, and immunofluorescence assays, respectively.

**Results:** The lentiviral vectors were constructed successfully. Immunofluorescence assays revealed the fluorescence intensity of α-tubulin to be decreased and α/β-tubulin to be increased in the mutation *MTUS1* group. The fluorescence intensity of PAR6 was higher and morphology of the daughter cells in the mutation group was different from the wild type group. The phosphorylation of Rac1/Cdc42 in the mutation group was significantly lower than in the wild type group.

**Conclusions:** A *de novo* mutation in *MTUS1* decreased the stability of microtubules and increased cell polarity via the Rac1/Cdc42 pathway, which may partly elucidate the mechanism underlying cellular protection in NVM.

## Introduction

Non-compaction of ventricular myocardium (NVM) is a structural abnormality of the left ventricular myocardium that is accompanied by severe clinical symptoms and a poor prognosis with no currently available effective prevention and therapeutic methods (1–4). NVM is diagnosed based on the ratio of the thickness of the non-compact endocardial layer to the thickness of the compact epicardial layer being >2.0 on echocardiograph (5). In the majority of patients, NVM is associated with genetic disease, particularly neuromuscular disorders and chromosomal defect (6, 7). The polarity of myocardial cells was recently reported to play an important role in the development of NVM (8, 9)_._ However, the underlying molecular mechanisms regulating cell polarity during early cardiac development and trabecular formation remain poorly understood.

Polarity is one of the basic processes that occur in living organisms (10). Cell polarity is the result of asymmetrical organization of cell membrane proteins and cell contents and can influence cell fate and specialized functions, such as migration, development, and proliferation (11). Cell polarity is controlled by Rho GTPase family members, the Par polarity complex, and cytoskeleton (12). Microtubules are a component of the cytoskeleton, found in eukaryotic cells, and formed by the polymerization of a dimer of two globular proteins, α, and β tubulin. These tubular polymers of tubulin are highly dynamic and stabilize the cell structure, transport intracellular substances, and mediate cell movement. The Rho GTPase family consists of six subfamilies: Rho, Rac, Cdc42, Rnd, RhoBTB, and RhoT/Miro (13)_._ The Rho GTPase family mediates the formation of the Par polarity complex, which causes cell polarity. The PAR proteins PAR3, PAR6, and aPKC localize to the anterior cortex, where PAR1 and PAR2 localize to the posterior pole and have essential functions in the first asymmetric division (14). Polar proteins are transported to the cell membrane through microtubule dynamic balance, thus forming polar protein complexes (15). In cardiac development, disruption of the cell polarity complex by targeted gene mutations results in aberrant mitotic spindles, loss of polarized cardiomyocyte division, and loss of normal myocardial trabeculation (9).

Microtubule-associated tumor suppressor 1 (MTUS1) encodes the microtubule-associated protein ATIP3, which cooperates with type-2 angiotensin II receptor (AGTR2) to inhibit extracellular signal-regulated kinase 2 (ERK2) activation and cell proliferation, which are closely related to cell division and migration (16, 17). A recently published study showed that MTUS1 knock-out mice developed spontaneous heart hypertrophy (18), suggesting that MTUS1 may affect cardiovascular system development.

However, the specific mechanisms underlying these processes have not yet been elucidated. Our team discovered a *de novo* mutation in *MTUS1*, c. 2617A->C (rs187103704), in a rare NVM family, that is likely associated with the mechanism underlying NVM. Therefore, the aim of this study was to investigate the association between a *de novo* mutation in *MTUS1* with NVM and to explore the potential mechanisms underlying this association. The current findings may help understand the genetic basis of NVM development, provide a theoretical basis for genetic counseling, prenatal diagnosis, and early intervention, and facilitate the development of new strategies for personalized medicine.

## Materials and Methods

### Subjects

A rare NVM family pedigree was discovered at the Children's Hospital of Chongqing Medical University. Blood samples were collected from the propositus and her family (sister, mother and aunt) for DNA extraction and whole exome sequencing (WES) (Deyi Oriental Translational Medicine Research Center, China). The original WES data were analyzed to confirm the biological relationships between the daughters, their mother and their aunt. First, the mutations identified by WES were selected by bioinformatics analysis and then functional predictions were made by making comparisons using Genebank, including the UCSC Genome Browser (http://genome.ucsc.edu/), GENECARDS (https://www.genecards.org/), the NCBI database (https://www.ncbi.nlm.nih.gov/), UNIPROT (https://www.uniprot.org/), and STRING (https://string-db.org/). Then the well-conserved mutations that caused amino acid polarity changes in important functional domains were screened as possibly pathogenic for NVM. The mutations were then identified by Sanger sequencing, with the Chromas software used for data analysis.

### Cell Culture and Transfections

HEK-293 cells, a classic cell line used in cell biology and gene research, were maintained at 37°C in a humidified atmosphere with 5% CO_2_ in Dulbecco's Modified Eagle Medium (Hyclone) containing 4.5 g/l glucose, 10% fetal bovine serum (Hyclone), and 100 mg/ml penicillin/streptomycin (19). Plasmosin (55 μl; InvivoGen, ant-mpt) was added to 550 ml complete medium to avoid mycoplasma contamination. *MTUS1* has multiple different isoforms with distinct functions. ATIP3 isoform (encoded by *MTUS1* gene), which expresses most in heart, has been studied most extensively. This isoform is known to play an important role in microtubule functions. We avoided selecting truncations or short isoforms which contain the mutant site of *MTUS1*, and we chose full length genes to construct lentiviruses, in order to avoid that the mutation site may affect the function of different functional domains through interaction. Lentiviruses containing GFP and FLAG-tags were completed by Genechem (China). To co-transfect with lentiviruses, HEK-293 cells were plated at a density of 2 ×10^5^ cells/5 ml in T25 culture flasks. Media were replaced with fresh media daily. Once the cells reached a density of about 40–50%, they were co-transfected with mutation variant, wild type, and vector lentiviruses at an MOI of 5. A total of 12 h after transfection, lentivirus-containing medium was replaced with fresh medium.

### *MTUS1* Expression Based on Real-Time PCR

At 24 h post-transfection, total RNA was extracted from HEK-293 cells using a TRIzol Reagent kit (Ambion) per the manufacturer's instructions and quantified by spectrophotometry at 260 nm. The mRNA was reversely transcribed using the Prime Script RT reagent kit containing gDNA Eraser (Takara, NO: RR047A) as described (20). The primers used for amplification were as follows: MTUS1, GAGCTGAGCACTTACAGCAACAA (forward) and TTCAACTGCATTAAGAGCTGTAA (reverse); and β-actin, CTCTTCCAGCCTTCCTTCCT (forward) and AGCACTGTGTTGGCGTACAG (reverse). The mRNA levels were normalized using β-actin as a housekeeping gene. Each experiment was repeated at least three times.

### Immunofluorescence Staining for Tubulin and PAR6

Cells plated on coverslips that were 30–40% confluent were fixed in an ice-cold 4% formaldehyde solution for 20 min prior to incubating for 15 min at room temperature with 0.05% Triton-X100 (21). The slides were then blocked with normal goat serum for 30 min. Slides were washed three times for at least 3 min each time after each incubation and then incubated overnight at 4°C with mouse anti-α-tubulin antibodies (Santa Cruz, USA, sc-5286, 1:50), rabbit anti-α/β-tubulin antibodies (Cell signaling, USA, Cat:2148,1:50), or rabbit anti-PAR6 antibodies (Abcam, USA, ab49776, 1:200). After washing three times the following day, cells were incubated for 60 min at room temperature with either Cy-3-conjugated anti-mouse antibodies or Cy-3-conjugated anti-rabbit antibodies (Seville, wuhan, 1:250) and then washed three times. The cell nuclei were identified by staining with DAPI (Roche, 10236276001, 5 μg/ml) for 15 min at room temperature. Coverslips were mounted on glass slides using DAPI Fluoromount-GTM (YEASEN, 36308ES11, Shanghai) and examined by confocal microscopy. Image analysis was performed using NIH and Image J software. Each experiment was repeated at least three times.

### Western Blot Analysis of Rac1/Cdc42

Protein was extracted from cells in exponential growth. Total cellular protein was extracted on ice using 1 × lysis buffer (KeyGEN BioTECH, NO: KGP250) supplemented with protease inhibitor (Roche, Switzerland) and phosphatase inhibitor (Roche, Switzerland). The entire protein extraction was performed strictly on ice. Protein concentrations were measured with the Coomassie (Bradford) Protein Assay (KeyGEN BioTECH, China). Total protein, 150 μg per lane, was separated on 12% SDS-PAGE gels. The SDS-PAGE gels and PVDF membranes were cut into corresponding sizes according to the molecular weights of the target proteins. Proteins were then transferred onto 0.22-μm PVDF membranes. Non-specific bands were blocked with Tris-buffered saline and Tween 20 (TBST) containing 5% bovine serum albumen for 1.5 h at room temperature. Then the membranes were incubated with specific primary antibodies at 4°C overnight. The next day, the PVDF membranes were incubated with the corresponding secondary antibodies at room temperature for 1.5 h (19, 20). Proteins bound to the 0.22-μm PDVF membranes were detected using primary antibodies against β-actin (4A Biotech, China, 1:1000), Rac1/Cdc42 (Cell signaling, 4651, USA, 1:1000), phospho-Rac1/Cdc42 (Ser71) (Cell signaling, 2461, USA, 1:500), or OctA-Probe (FLAG-tagged proteins; Santa Cruz, sc-166384, 1:500). Secondary antibodies were goat anti-mouse IgG (Millipore, GGHL-90P, 1:10000) or goat anti-rabbit IgG (Millipore,GGHL-15P,1:10000). Each experiment was repeated at least five times.

### Statistical Data Analysis

Statistical analysis was performed in SPSS version 20. At least three grids were prepared for each experimental condition examined in this study. Results are expressed as mean ± standard deviation. Differences among groups were analyzed by one-way analysis of variance (ANOVA). All *P*-values were two sided. *P* < 0.05 was considered statistically significant.

## Results

### Gene Sequencing Outcomes in Patients in Pedigree With NVM

**Figure 1:**
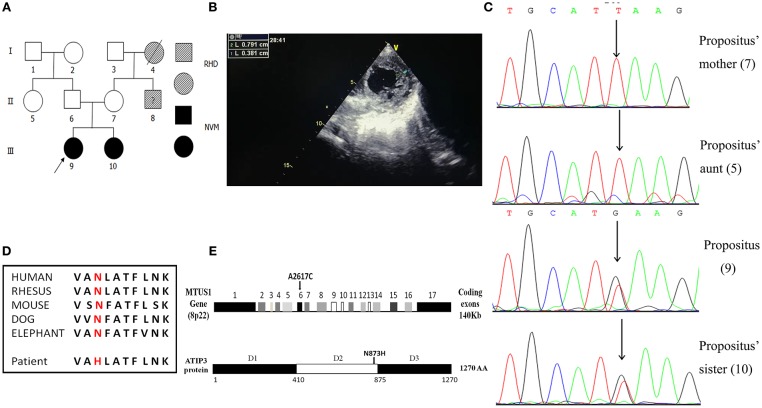
Familial pedigrees of NVM and information of the *de novo* mutation of *MTUS1* c. 2617A->C. **(A)** Pedigree showing individuals with non-compaction of ventricular myocardium (NVM) and Rheumatic Heart Disease (RHD). The arrow shows the index patient with NVM carrying the *MTUS1* mutation (c. 2617A->C). Squares, men; circles, women; black figures, individuals with NVM; oblique line figures, individuals with RHD; oblique line figures with a black question mark in the middle, suspicious RHD patient. **(B)** The echocardiographic image showing that the ratio of the thickness of the non-compact endocardial layer to the thickness of the compact epicardial layer was >2.0 (0.791/0.381). **(C)** Backward sequencing chromatogram of propositus and her family (sister, mother, and aunt). Parts of the nucleotide sequence are given below. The mutation site is pointed by an arrow (the position means c. 2617A->C). **(D)** Evolutionary conservation of c. 2617A->C mutation in all species. **(E)** Schematic representation of the structural organization shows human *MTUS1* gene and ATIP3 (encoded by *MTUS1* gene) protein regions. The *de novo* mutation of *MTUS1* c. 2617A->C located in exon 6. Position of the mutant amino-acid sequence was in D2. D1: domain 1, D2: domain 2, D3: domain 3.

In a rare patient with an NVM family pedigree, the propositus and her elder sister (Figure 1A) were definitively diagnosed with NVM based on clinical manifestations, echocardiography, and related examinations. Figure 1B shows the echocardiography analysis result of the propositus, which indicates that the ratio of the thickness of the non-compact endocardial layer to the thickness of the compact epicardial layer was 2.076 (0.791/0.381). Interestingly, their parents had normal clinical phenotypes. DNA samples from the two sisters and their mother and aunt were screened by WES and 32 mutation sites in 18 genes were selected for bioinformatics analysis and functional predictions. Other potential functional variants are shown in Supplementary Materials.

By Sanger sequencing analysis of the *MTUS1* gene in the two sisters, a heterozygous single nucleotide exchange at the position c. 2617A->C was identified (Figure 1C). In addition, we found that the c. 2617A->C mutation site was highly conserved among different species. The mutation changed the amino acid polarity from the hydrophobic Asn to the hydrophilic His at position 873 (N873H), leading to replacement of neutral amino-acids (amide side chains) by basic residues (Figure 1D). In schematic representation of the human *MTUS1* gene, the site of c. 2617A->C mutation located in exon 6, and in structural organization of ATIP3 protein (encoded by *MTUS1* gene), the position of the mutant amino-acid sequence was in D2 (Figure 1E), which decorates and stabilizes microtubules (22). It indicated that the mutant localized to an important functional domain. This mutation was present in the two sisters affected by NVM but absent from their mother and aunt. It means that a correlation between the NVM phenotype and the c. 2617A->C mutation was discovered in this NVM family. In a nutshell, the c. 2617A->C mutation in *MTUS1* is a *de novo* mutation and may be pathogenic for NVM.

### Lentiviral Vector Validation and Transfection

**Figure 2:**
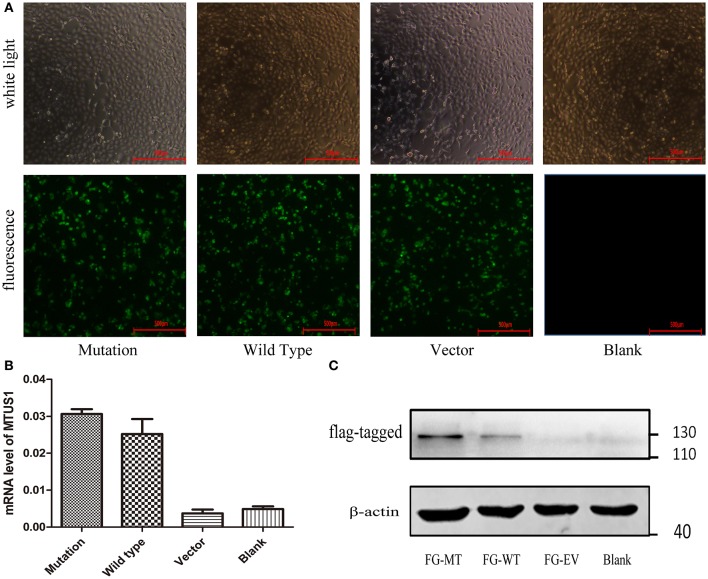
Lentiviral vector validation and transfection. **(A)** Co-transfect with lentiviruses containing GFP (c. 2617A->C mutation, wild type, vector, respectively), 48 h after transfection, cell state and transfection efficiency of each group were observed under ordinary microscope and fluorescence microscope. **(B)** After 48 h transfection, the *MTUS1* mRNA level expression was increased in the mutation and wild type group. **(C)** When GFP fluorescence occurs in mutation, wild type and vector group, the expression of flag-MTUS1 protein was detected to verify successful overexpression in HEK293 cells. FG-MT: flag-c. 2617A->C mutation, FG-WT: flag-wild type, FG-EV: flag-vector, Scale bar = 500 μm.

For HEK-293 cells, 48 h post-transfection, GFP and FLAG tag expressions by the lentiviral vector were measured based on fluorescence staining (Figure 2A) and western blot (Figure 2C). Meanwhile, *MTUS1* mRNA levels were increased based on real-time PCR (Figure 2B) in the mutation and wild type groups compared to the vector and blank groups. These results indicated transfection of the c. 2617A->C mutation *MTUS1* gene was successful.

### The c. 2617A->C Mutation Decreased α-tubulin Expression and Increased α/β-tubulin Expression

**Figure 3:**
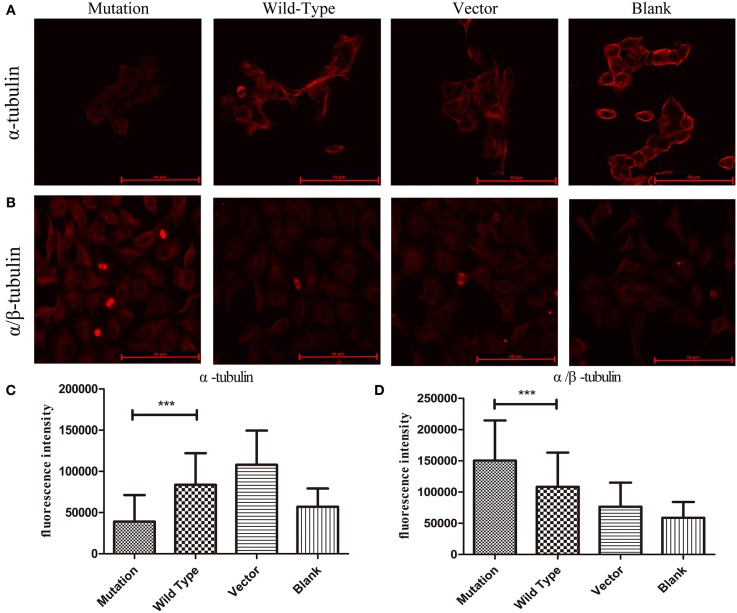
The stability of microtubules in HEK293 cells by immunofluorescence. **(A)** Images showing α-tubulin expression in four groups. The expression of α-tubulin in c. 2617A->C mutation group was significantly decreased than wild type group. **(B)** Images showing α/β-tubulin expression in four groups. Increased expression levels of α/β-tubulin in c.2617A->C mutation group were observed compared with wild type group. **(C)** Quantification of fluorescence intensity measurements of α-tubulin in four groups under the conditions described in **(A)**. **(D)** The quantification of fluorescence intensity of α/β-tubulin in four groups under the conditions described in **(B)**. ^***^P < 0.001; scale bar = 50 μm. Error bars show mean ± standard deviation in **(C,D)**.

The protein α-tubulin is a globular tubulin that serves as a subunit of microtubules to assess microtubule stability in this study. α/β-tubulin, the heterodimers have roles in the transportation functions of microtubule (21), which were examined to assess PAR protein transportation when *MTUS1* contained the c.2617A->C mutation. The fluorescence intensity of α-tubulin was found to be decreased in the c.2617A->C mutation group compared to the wild type group (*P* < 0.001, Figures 3A,C). Conversely, the fluorescence intensity of α/β-tubulin in the c. 2617A->C mutation group was significantly higher than in the wild type group (*P* < 0.001, Figures 3B,D).

### The c. 2617A->C Mutation Increased PAR6 Protein Expression

**Figure 4:**
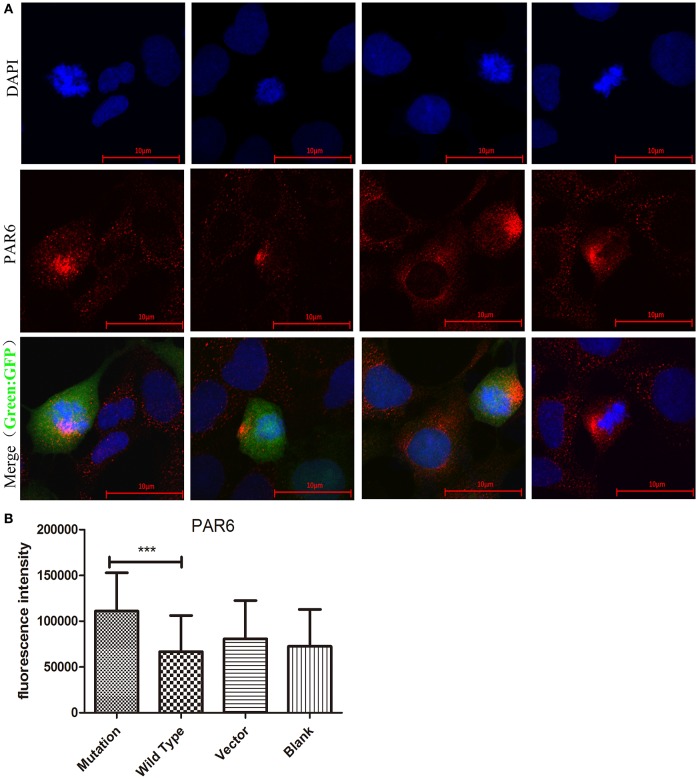
The c. 2617 A->C mutation increased PAR6 protein expression. **(A)** Images showing PAR6 protein (red) expression and location in four groups in cell division. Nuclei were stained by DAPI (blue). In Merged images, the location of PAR6 protein in c.2617A->C mutation group was abnormal, more partial to the side of daughter cells, compared with wild type group. **(B)** Quantification of fluorescence intensity of PAR6 in four groups under the conditions described in A. ^***^P < 0.001, Scale bar = 10 μm. Error bars show mean ± standard deviation.

PAR6, which acts as a polarity protein, is transported by microtubule heterodimers to one side of the cell membrane and forms polar PAR6-PAR3-aPKC complexes that affect cell polarity (23). The expression and location of PAR6 were analyzed in cells transfected with different recombinant lentiviruses to determine whether the c. 2617A->C mutation influences PAR6 protein. Our study showed that the fluorescence intensity of PAR6 protein was significantly increased in the c. 2617A->C mutation group compared to the wild type group (*P* < 0.001, Figure 4B), Interestingly, we found that the location of PAR6 protein in the mutation group was abnormal, where it was more partial to the side of daughter cells, compared to the wild type and blank groups (Figure 4A).

### The c. 2617A->C Mutation Regulated PAR6 Protein Expression in Association With Rac1/Cdc42 Signaling

**Figure 5:**
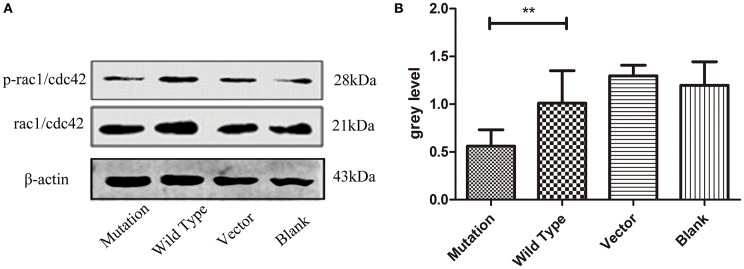
The c. 2617 A->C mutation regulated PAR6 protein expression in association with Rac1/Cdc42 signaling. **(A)** Western blotting analysis of phosphorylated Rac1/Cdc42 protein in four groups. Total Rac1/cdc42 is shown as a loading control. In cell division, the expression level of phosphorylated Rac1/Cdc42 was decreased in c. 2617A->C mutation than that in wild type group. **(B)** Quantification of western blot measurements of p-Rac1/Cdc42 level (total Rac1/Cdc42 as control) in four groups under the conditions described in A. ^**^P < 0.01; Error bars show mean ± standard deviation in **(B)**.

Rac1/Cdc42, the subfamily members of the Rho GTPase family, affect cell polarity, migration, and differentiation (24). Some studies have reported that Rac1/Cdc42 signaling plays a crucial role in adjusting the formation of PAR6-PAR3-aPKC complexes (25). Phosphorylated Rac1/Cdc42 inhibits GTP binding to Rac1/Cdc42, thereby weakening the downstream signal transduction pathway (26). Our study revealed that PAR6 protein levels were subject to Rac1/Cdc42 phosphorylation levels. Western blot analysis confirmed significantly lower phosphorylated Rac1/Cdc42 protein expression in the mutation group compared to the wild type and blank groups (*P* = *0.003, P* < 0.01, Figures 5A,B). These findings indicate that PAR6 protein expression in the mutation group was regulated by the phosphorylation level of Rac1/Cdc42.

### High Expression of PAR6 Protein in the c. 2617A->C Mutation-Carrying Cells Altered the Morphology of Daughter Cells

**Figure 6:**
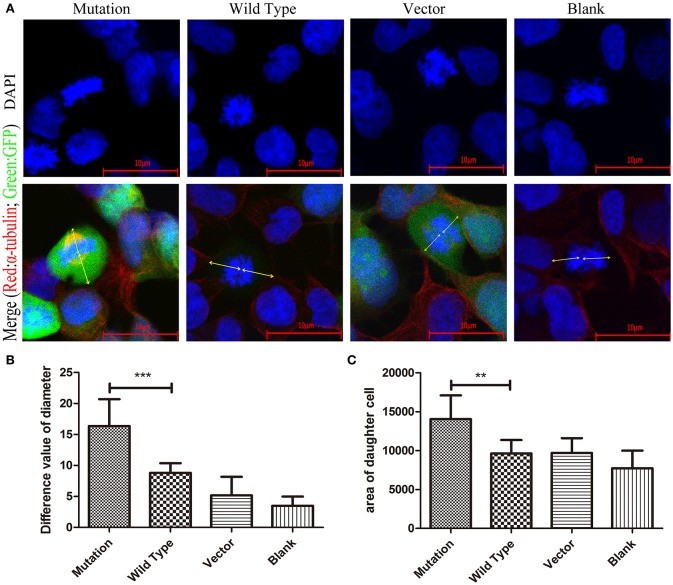
The morphology of daughter cells in HEK293 cells transfected with c. 2617A->C mutation. **(A)** In the merge images, the arrows (yellow) represent the distance from the spindle to the daughter cell membrane respectively at the stage of cell division. **(B)** Quantification of the distance from the spindle to the daughter cell membrane respectively at the stage of cell division in four groups under the conditions described in **(A)**. **(C)** The area of daughter cells in mutation group was obviously significantly larger than other groups. Blue, DAPI; Red, α-tubulin; Green, GFP. ^**^P < 0.01; ^***^P < 0.001. Scale bar = 10 μm. Error bars show mean ± standard deviation in **(B,C)**.

Cellular polarity cannot be established in the absence of the polarity protein complex. Disruption of the polarity protein complex results in aberrant mitotic spindle alignment and the loss of polarization of cells during cell division (27). Furthermore, changing the polarity of cells can lead to abnormal cell morphology and spindle localization (28). To further investigate the effect of increased expression of PAR6 protein in the mutation group on daughter cell morphology, we stained HEK-293 cells in each group for α-tubulin. We then examined the difference in the stained area in daughter cells and the distance between the spindle and cytoplasmic membrane in the daughter cells during cell division by immunofluorescence with confocal microscopy. The area in the daughter cells staining positive for α-tubulin in the mutation group was significantly larger than in the other groups (*P* < 0.001, Figures 6A,C). The distance between the spindle and cytoplasmic membrane of the daughter cells in the mutation group was significantly larger than in the wild type group (*P* < 0.01, Figures 4A,B). These results indicate that the c. 2617A->C mutation changed the morphology of the daughter cells after cell division by affecting the polarity of the cells.

## Discussion

NVM is a rare congenital cardiomyopathy resulting from an arrest in normal endomyocardial embryogenesis. The characteristic echocardiographic findings of NVM consist of multiple, prominent myocardial trabeculations, and deep intertrabecular recesses communicating with the left ventricular cavity (29, 30). The genetic causes and pathogenic mechanisms underlying this disease are largely unknown (31), though it has been described as an inherited cardiomyopathy with both familial and sporadic forms. Since the first genetic cause of NVM was identified as mutations in the X-linked TAZ gene, an increasing number of related genes have been found, including the sarcomere-encoding genes MYH7, ACTC, TNNT2, MYBPC3, TMP1, and TNNI3, Z-line protein-encoding ZASP/LDB3 gene, sodium channel gene SCN5A, and calcium-handling genes TAZ and LMNA (4, 32, 33). However, there have been few publications reporting an association between NVM and the gene encoding microtubules.

In the present study, we demonstrated a *de novo* mutation in *MTUS1*, a gene encoding microtubule-associated protein, caused decreased expression of α-tubulin, increased expression of α/β-tubulin heterodimer and PAR6 protein, enlarged the area of the daughter cells and the distance between the spindle and cytoplasmic membrane in daughter cells, and decreased levels of phosphorylated Rac1/Cdc42. These data are consistent with our hypothesis that the *de novo* c. 2617A->C mutation in *MTUS1* decreased the stability of microtubules and increased cell polarity, which might be through the Rac1/Cdc42 pathway, thus partly elucidating the protective mechanism in NVM.

The cytoskeleton is well known to be a dynamic three-dimensional structure that fills the cytoplasm of cells and is responsible for cell movement, cytokinesis, and the organization of organelles or organelle-like structures within cells. The major components of the cytoskeleton include microfilaments, microtubules, and intermediate filament systems. As one of the components of the cytoskeleton, microtubules may cause abnormalities in cardiac compacting during embryogenesis (34). In our study, we found that the fluorescence intensities in the mutated *MTUS1* group differed from the wild type group, indicating the microtubule stability in the mutated *MTUS1* group may have been destroyed. These results suggest the mutated *MTUS1* may alter myocardial densification by affecting microtubules during cardiac development.

Cell polarity refers to the unequal distribution of some cytoplasmic components in a cell in a certain spatial order, thus forming a concentration gradient of various intracellular components. Cellular biologic functions related to cell polarity include asymmetric cell division, migration, and proliferation. Some studies have found cardiomyocyte polarity to be disordered and cardiomyocyte morphology abnormal in heart slices from NVM model mice compared with normal heart sections, suggesting destruction of myocardial cell polarity may be involved in the occurrence of myocardial insufficiency during cardiac development (35, 36). The presence of cell polarity is inseparable from the formation of polar protein complexes and regulation of the Rho GTPase family, which are key regulatory factors associated with the cytoskeleton or microtubule stability (37, 38). The polar protein is transported from the cell cytoplasm to one side of the cell membrane by microtubule heterodimers to form the polar protein complexes that mediate polar cellular division. In the present study, we demonstrated that the *de novo* c. 2617A->C mutation in *MTUS1* increased cell polarity and concurrently decreased levels of phosphorylated Rac1/Cdc42. In addition, we found expression of polar protein PAR6, one of the members of the front-end polar protein complex, enhanced cell division, and located on the side of cell in the mutant group during cell division. These outcomes suggest that mutation of *MTUS1* increased polarity of HEK-293 cells by first lowering the phosphorylation level of Rac1/Cdc42 and then increasing expression of the polar protein PAR6, which is a downstream target gene of Rac1/Cdc42.

Biological functions related to cell polarization include asymmetric cell division and cell migration. However, cellular contents are unequally distributed when cell polarity is present (39). In this study, we found the area in daughter cells staining for α-tubulin and the distance between the spindle and cytoplasmic membrane of the daughter cells during cell division in the mutant group were obviously significantly larger than in other groups. It is suggested that the HEK-293 cells with the c. 2617A->C mutation underwent more asymmetric division than the wild type group due to changes in PAR6 protein levels. Furthermore, these results further suggest the *de novo* c. 2617A->C mutation in *MTUS1* promotes cell polarity in patients with NVM during cardiac development, increasing resistance to the disorder of adult cardiomyocytes that will occur in the development of NVM.

Although the present study was conducted in a cell culture system, the data had clinical relevance because the acquired mutation studied originated from a clinical patient. However, future studies are warranted to verify the effect of the *de novo* c. 2617A->C mutation in *MTUS1* on development of NVM and the underlying mechanism(s) using animal models and living human tissue. To date, there are no well-established animal models of NVM. Furthermore, it is prohibited to use living human tissue for ethical reasons. To overcome this challenge, we have already obtained the patient urine cells and generated induced pluripotent stem cell-derived cardiomyocytes, which carry the same genetic background as the patient (data not published). Next, to alleviate the phenotype of NVM mice model, the adeno associated virus (AAV) vectors containing mutation *MTUS1* will be injected into the pregnant mice of NVM model. Through these technologies, we will elucidate the roles of the *MTUS1* mutation in the NVM pathogenesis, as well as the underlying mechanism(s).

## Conclusion

Taken together, the data from the present study demonstrate that the Rac1/Cdc42 pathway might be involved in the control of cell polarity by the *de novo* c. 2617A->C mutation in *MTUS1*, thus may promote densification of the myocardium and reduce the occurrence of myocardial densification arrest. Therefore, the *de novo* c. 2617A->C mutation in *MTUS1* may be protective during cardiac development and may decrease the risk of NVM. This is the first study assessing the effects of *MTUS1* gene polymorphisms on cell polarity in NVM. These findings may help understand the genetic basis of cell polarity and provide insight for developing new approaches in the pathogenesis of NVM and reducing the prevalence of NVM through early gene intervention.

## Ethics Statement

This study was approved by the Institutional Review Board of Children's Hospital of Chongqing Medical University, China (Approval Notice 24/2015) and abided by the ethical principles outlined in the Declaration of Helsinki.

## Author Contributions

All authors contributed substantially to the conception and design of the study, and to the critical review of the manuscript. NO collected the samples and evaluated the WES result. YZ did the WES data analysis. XB completed the whole basic experiment and wrote the manuscript. LL, JT, XH, and TL reviewed the manuscript. All authors read and approved the final manuscript.

### Conflict of Interest Statement

The authors declare that the research was conducted in the absence of any commercial or financial relationships that could be construed as a potential conflict of interest.

## Abbreviations

NVM: non-compaction of ventricular myocardium
MTUS1: Microtubule-associated tumor suppressor 1
AGTR2: type-2 angiotensin II receptor
ERK2: extracellular signal-regulated kinase 2
WES: whole exome sequencing
ANOVA: one-way analysis of variance
RHD: rheumatic heart disease.
