# Supplementary material for: A de novo Mutation in the MTUS1 Gene Decreases the Risk of Non-compaction of Ventricular Myocardium via the Rac1/Cdc42 Pathway
Source: Front Pediatr. 2019 Jul 2;7:247. doi: 10.3389/fped.2019.00247 (PMC6626910; doi:10.3389/fped.2019.00247)
Supplement: Supplementary file 1 [file Data_Sheet_1.pdf]

Genetic mutations and benign polymorphisms:

Gene	chr location	Nucleic acid alteration	Amino acid alteration	rs Number	MAF	homozygous /heterozygous	Correlation with disease	Correlation statement	PubMed number	gene function or associated disease phenotype
TTN	chr2:179634600	c.8708C>T(E37)	p.2903,C>Y			heterozygous	possibly related	Predict deleterious		congenital myopathy, premature, with cardiomyopathy: autosomal recessive inheritance(AR); dilated cardiomyopathy 1G
	chr2:179518001	c.38755C>T(E198)	p.12919,A>T			heterozygous	possibly related	Predict deleterious		
	chr2:179576843	c.26763C>G(E94)	p.8921,W>C			heterozygous	possibly related	Predict deleterious		
congenital myopathy, premature,with cardiomyopathy：autosomal recessive inheritance(AR); ptosis; abnormal development in athletics; dilated cardiomyopathy; sudden death; Serum creatine phosphokinase elevation; Systemic myasthenia; facioplegia; arhythmia<br>dilated cardiomyopathy 1G：autosomal dominant(AD); dilated cardiomyopathy										
TPM1	chr15:63353080	c.505C>G(E5)	p.169,L>V			heterozygous	possibly related	Predict deleterious		dilated cardiomyopathy 1Y
dilated cardiomyopathy 1Y： AD; Congestive cardiac failure; dilated cardiomyopathy; ventricular tachycardia										
CTNNA3	chr10:68139090	c.1552C>G(E12)	p.518,V>L			heterozygous	possibly related	Predict deleterious		Arrhythmic right ventricular cardiomyopathy 13,AD
Arrhythmic right ventricular cardiomyopathy 13： AD; ventricular tachycardia; bundle branch block alternans										
FKTN	chr9:108398794	m.2851C>T(E11)	noncoding region	rs79589823	0.004	homozygous	unkown	mutation of UTR may affect translation of protein		dilated cardiomyopathy 1X ,AR
dilated cardiomyopathy 1X：AR; dilated cardiomyopathy; muscle weakness proximal										
AGBL1	chr15:86800251	IVS7+2T>G	splice site	rs138185152	0.002	heterozygous	Relatively high	The mutation is at the classical shear site		Fuchsdystrophia endothelialis corneae,AD
TGFBR2	chr3:30713619	c.944C>T(E4)	p.315,T>M	rs34833812	0.003	heterozygous	Relatively high	Association with diseases	9590282<br>17344846	Loeys-Dietz syndrome type 2 AD
ATP7B	chr13:52524268	c.2605C>T(E11)	p.869,G>R	rs191312027	0.001	heterozygous	Relatively high	Association with diseases	15952988	hepatolenticular degeneration,AR

ADCK3	chr1:227172643-227172644	IVS13+1 to IVS13+2:insert T	splice site			heterozygous	Relatively high	significant effect on mRNA and protein sequences		spinocerebellar ataxia type 9 Primary coenzyme Q10 deficiency type 4,AR
ABCG8	chr2:44101543-4 4101544	IVS9-3 至 IVS9-2: insert T	splice site			heterozygous	Relatively high	significant effect on mRNA and protein sequences		cholecystopathy type 4; sitosteremia,AR
ALS2	chr2:202588160	c.3517C>T(E22)	p.1173,E>K	rs41309046	0.006	heterozygous	possibly related	Predict deleterious		Primary lateral sclerosis in adolescents,AR; Infantile infant with progressive spastic paraplegia,AR; Ascending hereditary spastic paralysis in infants,AR
RYR1	chr19:39078032	c.15089G>A(E106)	p.5030,R>H			heterozygous	possibly related	Predict deleterious		malignant hyperthermia type 1,AD; central core disease,AD,AR; Microaxial hollow myopathy with extraocular palsy,AR
LFNG	chr7:2565976	c.920G>A(E6)	p.307,R>H	rs201791522	0.001	heterozygous	possibly related	Predict deleterious		Spinal rib dysplasia type 3
HGF	chr7:81336669	c.1553A>G(E14)	p.518,I>T			heterozygous	possibly related	Predict deleterious		epicophosis type 39,A R
	chr7:81386586	c.401C>T(E4)	p.134,R>H			heterozygous	possibly related	Predict deleterious		
USH2A	chr1:216172258	c.6628G>C(E34)	p.2210,P>A	rs192115090	0.002	heterozygous	possibly related	Predict deleterious		Yossi's syndrome type IIA ,AR; retinal pigment degeneration type 39 型,AR
PEPD	chr19:34003549	c.151C>G(E2)	p.51,G>R			heterozygous	possibly related	Predict deleterious		prolidase deficiency,AR

DGUOK	chr2:74166105	c.211C>T(E2)	p.71,P>S	rs184770596		heterozygous	possibly related	Predict deleterious		Mitochondrial DNA deletion syndrome type 3 (liver,brain) ,AR
DARS2	chr1:173799801	c.298G>A(E4)	p.100,A>T			heterozygous	possibly related	Predict deleterious		leukoencephalopathy, AR
FLNC	chr7:128482959	c.2501C>T(E16)	p.834,T>M	rs75133741	0.024	heterozygous	possibly related	Predict deleterious		myofibromyopathy type 5,AD
VCAN	chr5:82816856	c.2731A>C(E7)	p.911,S>R			heterozygous	possibly related	Predict deleterious		Wagner syndrome,AD
C6	chr5:41161953	c.1300T>C(E10)	p.434,I>V	rs117445335	0.004	heterozygous	possibly related	Predict deleterious		Complement 6 deficiency
POT1	chr7:124464107	c.1421C>G(E18)	p.474,C>S			heterozygous	possibly related	Predict deleterious		Familial malignant cutaneous melanoma susceptibility type 10,AD
WDR19	chr4:39216240	c.910G>A(E10)	p.304,V>I	rs75964850	0.031	heterozygous	possibly related	Predict deleterious		Asphyxiating thoracic dysplasia type 5,AR; cranioectodermal dysplasia type 4 ,AR ; Kidney disease type 13,AR
SLC2A2	chr3:170715711	c.1556C>T(E11)	p.519,G>E	rs147959014	0.005	heterozygous	possibly related	Predict deleterious		Fanco ni-Bickel syndrome), AR
RNASEH2B	chr13:51530530	c.859G>T(E11)	p.287,A>S	rs144408326	0.005	heterozygous	possibly related	Predict deleterious		Aicard i-Goutieres syndrome type 2,AR
ASS1	chr9:133333837	c.224C>T(E5)	p.75,P>L			homozygous	possibly related	Predict deleterious		urea cycle disorder /citrullinemia type I,AR
PHKA1	chrX:71904407	c.478C>T(E5)	p.160,D>N	rs201601894		heterozygous	possibly related	Predict deleterious		Glycogen accumulation type IXd,XR
ABCA3	chr16:2334870	c.3613C>T(E24)	p.1205,G>R	rs549977217	0	heterozygous	possibly related	Predict deleterious		pulmonary alveolar proteinosis type 3 AR

IL12RB1	chr19:18171942	c.1781C>T(E15)	p.594,G>E	rs370238890	0.002	heterozygous	possibly related	Predict deleterious		immunodeficiency 30
IFT140	chr16:1574615	c.3079C>T(E24)	p.1027,G>R	rs137995818	0.007	homozygous	possibly related	Predict deleterious		MAINZER-SALDINO syndrome,AR
CUBN	chr10:17153023	c.910C>T(E9)	p.304,E>K	rs78201384	0.009	heterozygous	possibly related	Predict deleterious		megaloblastic anemia type 1 ,AR
BFSP1	chr20:17475569	c.773T>C(E8)	p.258,N>S	rs150798461	0.008	heterozygous	possibly related	Predict deleterious		Cortical cataract in adolescents
FREM1	chr9:14770803	c.467A>G(E3)	p.156,V>A	rs190763692	0.001	heterozygous	possibly related	Predict deleterious		Mani toba Oculotric hoanal syndrome,AR
TBXA2R	chr19:3595747	c.971C>T(E3)	p.324,S>N			heterozygous	possibly related	Predict deleterious		hemorrhagic Disorders, AD
HDAC6	chrX:48681922	c.3113A>G(E25)	p.1038,Q>R			heterozygous	possibly related	Predict deleterious		achondroplasia syndrome,XD
NBN	chr8:90965508	c.1809G>T(E11)	p.603,F>L	rs192236678	0.001	heterozygous	possibly related	Predict deleterious		aplastic anemia; acute lymphoblastic leukemia; Nijmegen breakage syndrome,AR
ALG3	chr3:183960348	c.1127G>A(E9)	p.376,P>L	rs79144888	0.003	heterozygous	possibly related	Predict deleterious		congenital disorders of glycosylation type 1D,AR
SLC1A3	chr5:36677004	c.578A>G(E5)	p.193,N>S			heterozygous	possibly related	Predict deleterious		Intermittent ataxia type 6
SPECC1L	chr22:24743090	c.2689C>T(E10)	p.897,P>S	rs148229290	0.005	heterozygous	possibly related	Predict deleterious		oblique facial cleft type 1
KIF1C	chr17:4927256	c.3122G>A(E23)	p.1041,R>Q	rs201887996	0.001	heterozygous	possibly related	Predict deleterious		spastic spinal paralyses type 2,AR

DCC	chr18:49867165	c.8A>G(E1)	p.3,N>S	rs117282798	0.001	heterozygous	possibly related	Predict deleterious		mirror movements type 1 ,AD
ADAMTS17	chr15:100589135	c.2518C>T(E18)	p.840,D>N	rs147316982	0.003	heterozygous	possibly related	Predict deleterious		Weill-Marchesani syndrome,AR
EBP	chrX:48385649	c.445A>G(E4)	p.149,I>V			heterozygous	possibly related	Predict deleterious		Conradi-Hunermann-Happlesyndrome,XD
COL4A1	chr13:110866312	c.195C>G(E3)	p.65,M>I			heterozygous	possibly related	Predict deleterious		familial porencephaly type1,AD; Hereditary vascular disease with nephropathy、Microaneurysms and muscle spasms,AD; Axenfeld-Rieger dieases,AD;
FLT3	chr13:28622544	c.1073T>A(E9)	p.358,D>V	rs34172843	0.005	heterozygous	possibly related	Predict deleterious		acute lymphoblastic leukemia
FIG4	chr6:110056444	c.589C>T(E6)	p.197,R>C			heterozygous	possibly related	Predict deleterious		amyotrophic lateral sclerosis type11; Bilateral temporo-occipital polycerebellum malformations,AR; Yunis-Varon syndrome,AR
EDN1	chr6:12292615	c.103G>A(E2)	p.35,G>R	rs183694577	0.003	heterozygous	possibly related	Predict deleterious		Atrioventricular condyle syndrome type 3,AR; Solitary question mark ear,AD
GLDC	chr9:6606634	c.671C>T(E5)	p.224,R>H	rs28617412	0.031	heterozygous	possibly related	Predict deleterious		Nonketogenic hyperglycemia/ Glycine encephalopathy,AR
ATP2B3	chrX:152835144	c.3426T>G(E20)	p.1142,H>Q			heterozygous	possibly related	Predict deleterious		spinocerebellar ataxia 1,XR

CEP290	chr12:88505514	c.2174T>G(E21)	p.725,E>A	rs375038986	0.001	heterozygous	possibly related	Predict deleterious		Meckel syndrome type 4 ;Sen ior-Loken syndrome type 6,AR; Joubert syndrome type 5,AR;Bardet-biedl syndrome type14,AR; Leber's congenital amaurosis type10
POLH	chr6:43578382	c.1166G>A(E10)	p.389,R>H	rs564211260	0	heterozygous	possibly related	Predict deleterious		xeroderma pigmentosum ,AR
LHFPL5	chr6:35773782	c.335T>C(E1)	p.112,I>T	rs202178128	0.001	heterozygous	possibly related	Predict deleterious		AR, epicophosis 67
EXT2	chr11:44129444	c.281G>A(E2)	p.94,R>H	rs151228794	0	heterozygous	possibly related	Predict deleterious		hereditary multiple exostosis type2,AD
LHX3	chr9:139089401	c.979C>T(E6)	p.327,A>T	rs201356862	0.003	heterozygous	possibly related	Predict deleterious		Combined pituitary hormone deficiency type3,AR
TCTN2	chr12:124189171	c.1702C>G(E15)	p.568,L>V	rs371239697	0	heterozygous	possibly related	Predict deleterious		Meckel syndrome type 8
	chr12:124179439	c.1147C>T(E10)	p.383,H>Y	rs372235872	0	heterozygous	possibly related	Predict deleterious		
AIRE	chr21:45712994	c.1214C>A(E10)	p.405,P>Q			heterozygous	possibly related	Predict deleterious		autoimmune polyendocrine syndrome type1 ,AD,AR
ADAR	chr1:154573911	c.322T>C(E2)	p.108,M>V			heterozygous	possibly related	Predict deleterious		Aicard i-Goutieres syndrome type6,AR; dyschromatosis symmetrical hereditaria,AD
GFI1B	chr9:135866403	c.821G>A(E6)	p.274,R>Q			heterozygous	possibly related	Predict deleterious		Thrombocytic hemorrhagic disease type 17,AD
SUMO4	chr6:149721667	c.140A>G(E1)	p.47,Y>C			heterozygous	possibly related	Predict deleterious		insulin-dependent diabetes mellitus type5

AGT	chr1:230838915	c.1430C>T(E5)	p.477,R>H	rs146284519	0.001	heterozygous	possibly related	Predict deleterious		Essential hypertension; Renal tubular dysplasia, AR; pre-eclampsia,AD
DFNA5	chr7:24756983	c.587G>A(E5)	p.196,T>M	rs183361282	0.001	heterozygous	possibly related	Predict deleterious		epicophosis 5,AD
PYGM	chr11:64514397	c.2111T>C(E17)	p.704,Y>C			heterozygous	possibly related	Predict deleterious		McArd le dieases,AR
CASR	chr3:122002576	c.1775A>G(E7)	p.592,N>S	rs117375173	0.009	heterozygous	possibly related	Predict deleterious		idiopathic generalized epilepsy type 8; Familial hypocalcemia and hypercalcemia,AD; hypocalcemia,AD; Severe parathyroidism in neonates,AR
TLR3	chr4:186997925	c.152C>G(E2)	p.51,T>R			heterozygous	possibly related	Predict deleterious		HPV2;HIV1
TTN	chr2:179634600	c.8708C>T(E37)	p.2903,C>Y			heterozygous	possibly related	Predict deleterious		Delayed tibial muscular dystrophy,AD; hereditary myopathy; Limb-girdle muscular dystrophy 2J,AR; familial hypertrophic cardiomyopathy 9
	chr2:179518001	c.38755C>T(E198)	p.12919,A>T			heterozygous	possibly related	Predict deleterious		
	chr2:179576843	c.26763C>G(E94)	p.8921,W>C			heterozygous	possibly related	Predict deleterious		
SETX	chr9:135203876	c.3109C>A(E10)	p.1037,D>Y	rs569757584		heterozygous	possibly related	Predict deleterious		Early onset ataxia with eye movement cannot;Hypoalbuminea type 2,AR; amyotrophic lateral sclerosis,,AD
NALCN	chr13:101733907	c.3856C>A(E34)	p.1286,V>L			heterozygous	possibly related	Predict deleterious		Low muscular tone in children,AR
SPRED1	chr15:38641670	c.630A>T(E6)	p.210,E>D			heterozygous	possibly related	Predict deleterious		Neurofibromatosis syndrome type1,AD

TCN2	chr22:31011772	c.938G>A(E6)	p.313,R>Q	rs576306465	0	heterozygous	possibly related	Predict deleterious		Vitamin B12 deficiency /TC II deficiency / Cobalamin transporter II deficiency,AR
EVC	chr4:5795410	c.1852G>A(E13)	p.618,G>S			heterozygous	possibly related	Predict deleterious		Weyers Cephalic and facial hypoplasia syndrome,AD;
EPHX1	chr1:226016558	c.128G>C(E2)	p.43,R>T	rs3738046	0.01	heterozygous	possibly related	Predict deleterious		Familial hypercholanemia; pre-eclampsia,AD
ITGA8	chr10:15701045	c.901T>A(E10)	p.301,I>F	rs375739322	0.002	heterozygous	possibly related	Predict deleterious		renal dysplasia,AR
MUC5B	chr11:1269442	c.11332G>A(E31)	p.3778,A>T	rs199966165		heterozygous	possibly related	Predict deleterious		Idiopathic Pulmonary Fibrosis,AD
MLPH	chr2:238419729	c.430C>T(E4)	p.144,R>W	rs138667878	0	heterozygous	possibly related	Predict deleterious		Grisc elli syndrome type3,AR
GNRHR	chr4:68610309	c.719C>T(E2)	p.240,R>Q	rs182531866	0.001	heterozygous	possibly related	Predict deleterious		Hypoglandular dysfunction type 7, AD
MEN1	chr11:64575505	c.527C>T(E3)	p.176,R>Q	rs607969	0.006	heterozygous	possibly related	Predict deleterious		multiple endocrine neoplasia type1,AD
CNTNAP2	chr7:146829495	c.1242C>G(E8)	p.414,H>Q			heterozygous	possibly related	Predict deleterious		Autism Pitt - Hopkinssyndrome1/ Cortical dysplasia with focal epilepsy syndrome
NCR3	chr6:31556928	c.522C>A(E4)	p.174,R>S	rs3179003	0.032	heterozygous	possibly related	Predict deleterious		Mild malaria, susceptible
CCDC50	chr3:191093285	c.883G>T(E6)	p.295,D>Y	rs75123867	0.005	heterozygous	possibly related	Predict deleterious		AD epicophosis type 44

GLIS2	chr16:4383447	c.272C>A(E2)	p.91,S>Y	rs200911856	0	heterozygous	possibly related	Predict deleterious		Kidney disease type 7
TPM1	chr15:63353080	c.505C>G(E5)	p.169,L>V			heterozygous	possibly related	Predict deleterious		Familial hypertrophic cardiomyopathy,AD
CWF19L1	chr10:101993090	c.1511T>A(E14)	p.504,D>V	rs117713471	0.007	heterozygous	possibly related	Predict deleterious		spinocerebellar ataxia 17,AR
EPX	chr17:56272524	c.794C>T(E6)	p.265,P>L	rs201161563	0	heterozygous	possibly related	Predict deleterious		Eosinophilic peroxidase and phospholipid deficiency,AR
KRT1	chr12:53073831	c.302C>A(E1)	p.101,G>V	rs147840212		heterozygous	possibly related	Predict deleterious		Curth-Macklin ichthyosis,AD; Epidermolytic hyperkeratosis annular ichthyosis,AD; Epidermolytic keratosis palmairs et plantaris,AD; Non - epidermal lax palmoplantar keratosis,AD; Palmoplantar keratosis striatum type 3
NEFH	chr22:29885554	c.1925C>T(E4)	p.642,T>M	rs117258406	0.004	heterozygous	possibly related	Predict deleterious		amyotrophic lateral sclerosis type 1,AD,AR
APC	chr5:112174614	c.3323A>G(E16)	p.1108,N>S	rs151286353	0	heterozygous	possibly related	Predict deleterious		familial adenomatous polyposis type1,AD; Hereditary desmofibromatosis, AD

ATXN7	chr3:63981215	c.1717A>G(E12)	p.573,I>V	rs3733124	0.01	heterozygous	possibly related	Predict deleterious		Spinocerebellar Ataxias type 7（ADCA II）,AD
MAPK8IP1	chr11:45924019	c.701A>T(E5)	p.234,D>V			heterozygous	possibly related	Predict deleterious		Non-insulin-dependent diabetes mellitus AD
NAGA	chr22:42459035	IVS6-7G>T	splice site	rs150693978	0.005	heterozygous	possibly related	mRNA splicing		Kanzaki dieases,AR;Schindler type I and typeIII,AR

SYNE1	chr6:152542702	IVS116-8A>C	splice site	rs187773880	0.005	heterozygous	possibly related	mRNA splice		
	chr6:152722413	c.6910C>T(E47)	p.2304,G>R	rs117184249	0.004	heterozygous	possibly related	Predict deleterious		spinocerebellar ataxia type 8 ,AR;Emery-Dreifuss type 4 ,AD
GYPA	chr4:145041750	IVS1-9A>C	splice site	rs45468202		homozygous	possibly related	mRNA splice		Malaria, susceptible
PRKDC	chr8:48701786	c.10683A>T(E75)	p.3561,S>R	rs8178232	0.007	heterozygous	unkown	unkown		immunodeficiency 26，With or without neurological abnormalities,AR
MUC17	chr7:100683336	c.8639C>T(E3)	p.2880,P>L	rs142042551	0.004	heterozygous	possibly related	Predict deleterious		
CENPT	chr16:67862706	c.1321G>C(E14)	p.441,P>A	rs188159138	0.002	heterozygous	possibly related	Predict deleterious		
KRT78	chr12:53242498	c.217G>A(E1)	p.73,P>S	rs139572939	0.003	heterozygous	possibly related	Predict deleterious		
SP2	chr17:45994116	c.679G>T(E3)	p.227,A>S	rs150853499	0.007	heterozygous	possibly related	Predict deleterious		
MICALCL	chr11:12315636	c.658G>A(E3)	p.220,G>R	rs150509319	0.003	heterozygous	possibly related	Predict deleterious		
RGS22	chr8:101076173	c.823C>T(E8)	p.275,E>K			heterozygous	possibly related	Predict deleterious		
KRT73	chr12:53007474	c.982T>G(E5)	p.328,K>Q			heterozygous	possibly related	Predict deleterious		
WNT2B	chr1:113057615	c.245G>A(E3)	p.82,R>Q	rs140445206	0.002	heterozygous	possibly related	Predict deleterious		
LRGUK	chr7:133932368	c.2044C>T(E17)	p.682,R>C	rs140175129	0.007	heterozygous	possibly related	Predict deleterious		
FRMPD2	chr10:49420083	c.1525C>T(E13)	p.509,A>T			heterozygous	possibly related	Predict deleterious		
OR5K4	chr3:98072930	c.233C>A(E1)	p.78,T>N	rs193142226	0.001	heterozygous	possibly related	Predict deleterious		

CENPN	chr16:81056395	c.567A>C(E7)	p.189,Q>H	rs138454126	0.003	heterozygous	possibly related	Predict deleterious		
ZBTB7B	chr1:154987750	c.716G>A(E4)	p.239,R>Q	rs553961442	0	heterozygous	possibly related	Predict deleterious		
C11orf30	chr11:76256929	c.3362G>A(E20)	p.1121,G>D			heterozygous	possibly related	Predict deleterious		
PIK3R3	chr1:46546408	c.121G>A(E3)	p.41,P>S	rs115181807	0.001	heterozygous	possibly related	Predict deleterious		
ZC3H12A	chr1:37946018	c.571G>A(E3)	p.191,V>M			heterozygous	possibly related	Predict deleterious		
C5orf48	chr5:125968261	c.110G>A(E2)	p.37,R>Q	rs147526168	0	heterozygous	possibly related	Predict deleterious		
POU2F2	chr19:42596250	c.1187G>A(E13)	p.396,S>L	rs372097777		heterozygous	possibly related	Predict deleterious		
ROPN1B	chr3:125694422	c.133T>C(E3)	p.45,S>P	rs187992194	0.003	heterozygous	possibly related	Predict deleterious		
KRTAP10-10	chr21:46057613-46057627	c.279(E1) to c.293(E1): missing CTGCT<5>CCTGT	missing non-frame shift	rs56249559		heterozygous	possibly related	Predict deleterious		
NCKAP5L	chr12:50189952	c.1691C>T(E8)	p.564,R>Q	rs576123127	0.001	heterozygous	possibly related	Predict deleterious		
KRTAP10-12	chr21:46117784	c.668C>T(E1)	p.223,P>L	rs556883214		heterozygous	possibly related	Predict deleterious		
GPR1	chr2:207041779	c.193A>G(E3)	p.65,F>L	rs1060384	0.005	heterozygous	possibly related	Predict deleterious		
CALD1	chr7:134618471	c.951G>A(E5)	p.317,M>I	rs117593246	0.003	heterozygous	possibly related	Predict deleterious		
PPP4R1	chr18:9583200	c.833G>A(E9)	p.278,A>V	rs182385365	0.001	heterozygous	possibly related	Predict deleterious		
PEG3	chr19:57328240	c.1570G>A(E9)	p.524,R>W			heterozygous	possibly related	Predict deleterious		
KRTAP1-5	chr17:39182945	c.463G>A(E1)	p.155,R>C	rs148850772	0.006	heterozygous	possibly related	Predict deleterious		

C11orf42	chr11:6231170	c.163C>T(E2)	p.55,Q>X(279)			heterozygous	Relatively high	nonsense mutation		
NRK	chrX:105168893	c.3182G>T(E19)	p.1061,G>V	rs376619055		heterozygous	possibly related	Predict deleterious		
OXNAD1	chr3:16344963	c.833C>A(E9)	p.278,T>N			heterozygous	possibly related	Predict deleterious		
CCDC51	chr3:48474284	c.770C>T(E4)	p.257,R>H	rs185891886	0.002	heterozygous	possibly related	Predict deleterious		
NLRP2	chr19:55501965-55501966	c.2633(E10) to c.2634(E10): insert C	p.878,N>Nfs13			heterozygous	Relatively high	cause a significant change in the protein sequence		
EML6	chr2:55181275	c.4468G>T(E31)	p.1490,V>F			heterozygous	possibly related	Predict deleterious		
MCM2	chr3:127336910	c.1999G>A(E12)	p.667,V>M	rs2307311	0.008	heterozygous	possibly related	Predict deleterious		
MPP4	chr2:202519559	IVS18+1C>T	splice site			heterozygous	Relatively high	The mutation is at the classical shear site		
FAM58BP	chr1:200182710-200182721	c.19(E1) to c.30(E1):missing GCCGGAGAGGAG	missing non- frame shift	rs565394962	0.039	heterozygous	possibly related	Predict deleterious		
NLRP4	chr19:56382266	c.2428C>T(E7)	p.810,R>C	rs143466082		heterozygous	possibly related	Predict deleterious		
CCDC88A	chr2:55582884	c.631T>C(E8)	p.211,I>V	rs201676947	0	heterozygous	possibly related	Predict deleterious		
LCE4A	chr1:152681680-152681681	c.129(E1) to c.130(E1):insert AGCTCTGGGGGCTGCTGT	insert non- frame shift	rs6143428		heterozygous	possibly related	Predict deleterious		
SHC4	chr15:49126970	c.1733C>T(E11)	p.578,G>D			heterozygous	possibly related	Predict deleterious		

PLCB2	chr15:40587463-40587463	c.1720(E17): missing A	p.574,S>Rfs27			heterozygous	Relatively high	cause a significant change in the protein sequence			
ARHGAP8	chr22:45244912-45244912	c.942(E11): missing G	p.315,A>Pfs54			heterozygous	Relatively high	cause a significant change in the protein sequence			
SHC1	chr1:154940975	c.746T>C(E4)	p.249,K>R			heterozygous	possibly related	Predict deleterious			
ATP8B2	chr1:154317149	c.2273G>A(E21)	p.758,R>H	rs118078368	0.001	heterozygous	possibly related	Predict deleterious			
DGCR6	chr22:18899156	c.617T>C(E5)	p.206,L>S			heterozygous	possibly related	Predict deleterious			
HEATR5B	chr2:37297483	c.817G>C(E7)	p.273,L>V			heterozygous	possibly related	Predict deleterious			
DGCR2	chr22:19050757	c.460G>A(E4)	p.154,R>W			heterozygous	possibly related	Predict deleterious			
TAAR9	chr6:132860014	c.586C>T(E1)	p.196,Q>X(153)	rs148058947	0	heterozygous	Relatively high	Nonsense mutations may lead to premature termination of protein translation			
CD97	chr19:14507189	c.382A>G(E5)	p.128,K>E	rs570748230	0.001	heterozygous	possibly related	Predict deleterious			
TTLL11	chr9:124632992	c.1787T>C(E7)	p.596,E>G	rs140709315	0.002	heterozygous	possibly related	Predict deleterious			
C8orf58	chr8:22458676	c.322C>T(E2)	p.108,L>F			heterozygous	possibly related	Predict deleterious			
ARHGAP19	chr10:99019287	c.712G>A(E5)	p.238,P>S	rs61751502	0.002	heterozygous	possibly related	Predict deleterious			
ZNF84	chr12:133633610	c.309A>T(E5)	p.103,R>S	rs202216892		heterozygous	possibly related	Predict deleterious			
OR6K6	chr1:158724634	c.29A>G(E1)	p.10,H>R	rs75906759	0.005	heterozygous	possibly related	Predict deleterious			

RRS1	chr8:67341743	c.377A>G(E1)	p.126,K>R	rs3739335	0.026	heterozygous	possibly related	Predict deleterious		
TXNDC11	chr16:11792171	c.917C>T(E7)	p.306,R>K	rs74801303	0.002	heterozygous	possibly related	Predict deleterious		
TXNRD3NB	chr3:126291298	c.89C>A(E3)	p.30,S>I	rs73195848	0.046	heterozygous	possibly related	Predict deleterious		
TET1	chr10:70406444	c.3958C>T(E4)	p.1320,R>W	rs149434944	0	heterozygous	possibly related	Predict deleterious		
OR6C76	chr12:55820104	c.67G>A(E1)	p.23,V>I	rs74092309	0.026	heterozygous	possibly related	Predict deleterious		
KCTD19	chr16:67333330	c.922C>T(E6)	p.308,E>K			heterozygous	possibly related	Predict deleterious		
ENTPD3	chr3:40453442	c.426G>T(E5)	p.142,M>I	rs200476681	0.001	heterozygous	possibly related	Predict deleterious		
	chr3:40457374	c.641C>A(E7)	p.214,T>N	rs201157099	0.001	heterozygous	possibly related	Predict deleterious		
LOC100996465	chrX:153151277-153151285	c.142(E5) to c.150(E5):missing 失 CCCGCAGGT insert GCCGCCCAGG	complex non-frameshift	rs143464562,rs59857608,rs199975679		homozygous	Relatively high	cause a significant change in the protein sequence		
PRAMEF6	chr1:13001312	c.371C>G(E3)	p.124,C>S	rs61779367		heterozygous	possibly related	Predict deleterious		
EIF3A	chr10:120820818	c.1145T>C(E8)	p.382,Y>C	rs149836727		heterozygous	possibly related	Predict deleterious		
KCNS3	chr2:18113615	c.1340G>A(E3)	p.447,R>Q	rs78213799	0	heterozygous	possibly related	Predict deleterious		
CSMD1	chr8:2857552	c.8131G>A(E53)	p.2711,R>W	rs562712934	0	heterozygous	possibly related	Predict deleterious		
PCDHAC2	chr5:140347787	c.1436A>G(E1)	p.479,Q>R	rs185216314	0	heterozygous	possibly related	Predict deleterious		
								Predict		

NAPB	chr20:23401975	c.65T>C(E1)	p.22,K>R	rs140977227	0.009	heterozygous	possibly related	deleterious		
CSMD3	chr8:113519035	c.4468C>T(E28)	p.1490,G>R			heterozygous	possibly related	Predict deleterious		
PEAK1	chr15:77471648	c.2621C>T(E5)	p.874,R>H	rs556459431	0	heterozygous	possibly related	Predict deleterious		
TBC1D3C	chr17:34590453	c.2A>G(E2)	p.1,M>T	rs539675313	0.005	heterozygous	Relatively high	mutation in initiation codon, Affect protein coding initiation site		
COPZ2	chr17:46111240	c.253C>T(E3)	p.85,A>T			heterozygous	unkown	unkown		
SLITRK5	chr13:88327997	c.354G>T(E2)	p.118,Q>H	rs149464166	0.001	heterozygous	possibly related	Predict deleterious		
AKAP2	chr9:112899278	c.1028T>C(E2)	p.343,L>P			heterozygous	possibly related	Predict deleterious		
KIR2DL3	chr19:55250979	c.61C>A(E2)	p.21,P>T	rs143258060		heterozygous	possibly related	Predict deleterious		
ERC1	chr12:1192664	c.1004G>A(E2)	p.335,R>Q	rs74740082	0.003	heterozygous	possibly related	Predict deleterious		
SLC25A33	chr1:9642548	c.955C>T(E7)	p.319,R>C	rs149047690	0.001	heterozygous	possibly related	Predict deleterious		
	chr1:9642390	c.797C>G(E7)	p.266,T>S	rs201307204	0.001	heterozygous	possibly related	Predict deleterious		
ZNF195	chr11:3380998	c.1171C>T(E5)	p.391,E>K	rs189086448	0.005	heterozygous	possibly related	Predict deleterious		
PRAMEF13	chr1:13448199	c.1276C>T(E4)	p.426,D>N	rs143762964		heterozygous	possibly related	Predict deleterious		
MAP4K3	chr2:39559095	c.493T>C(E8)	p.165,I>V			heterozygous	possibly related	Predict deleterious		
ZNF192	chr6:28119650	c.587G>A(E4)	p.196,R>H			heterozygous	possibly related	Predict deleterious		
FAM118A	chr22:45723947	IVS5+3A>G	splice site	rs74676649		heterozygous	possibly related	mRNA splice		
								Predict		

GTF2IRD2	chr7:74225480	c.755T>C(E10)	p.252,H>R	rs200821956	0.031	heterozygous	possibly related	deleterious		
CA10	chr17:49726596	c.581G>C(E7)	p.194,P>R	rs78297325	0.004	heterozygous	possibly related	Predict deleterious		
RC3H1	chr1:173915664	c.2911G>C(E16)	p.971,Q>E			heterozygous	possibly related	Predict deleterious		
PAGE5	chrX:55249049	IVS3-5T>C	splice site	rs2148982		homozygous	possibly related	mRNA splice		
SRRM2	chr16:2818097	c.7568C>G(E11)	p.2523,A>G	rs117824484	0.005	heterozygous	possibly related	Predict deleterious		
SRRM5	chr19:44117271	c.998G>T(E1)	p.333,R>I	rs12608798		heterozygous	possibly related	Predict deleterious		
PUS7L	chr12:44130360	c.1549G>C(E7)	p.517,Q>E	rs201646830	0	heterozygous	possibly related	Predict deleterious		
APOBEC3B	chr22:39382376	c.528A>C(E4)	p.176,E>D	rs144209680	0.007	heterozygous	possibly related	Predict deleterious		
TSSC4	chr11:2424220	c.357C>A(E2)	p.119,N>K	rs142339475		heterozygous	possibly related	Predict deleterious		
GABRB2	chr5:160757907	c.1060G>A(E9)	p.354,R>C	rs41298406	0.001	heterozygous	possibly related	Predict deleterious		
CD14	chr5:140011867	c.702C>T(E2)	p.234,M>I	rs74587733	0.011	homozygous	possibly related	Predict deleterious		
SPATA31A3	chr9:40706227	c.3884G>A(E4)	p.1295,R>Q			heterozygous	possibly related	Predict deleterious		
TMTC1	chr12:29709853	c.1613T>C(E10)	p.538,Q>R	rs190598821	0.002	heterozygous	possibly related	Predict deleterious		
SVIL	chr10:29777559	c.3041T>A(E21)	p.1014,Y>F	rs149858297	0.005	heterozygous	possibly related	Predict deleterious		
	chr10:29754535	c.4844G>A(E32)	p.1615,A>V	rs17694739	0.049	heterozygous	possibly related	Predict deleterious		
FYTTD1	chr3:197483345	c.74C>T(E3)	p.25,P>L			heterozygous	possibly related	Predict deleterious		
								Predict		

SLC16A6	chr17:66267508	c.793T>G(E6)	p.265,M>L	rs138318896		heterozygous	possibly related	deleterious		
TENM4	chr11:78380625	c.6765C>T(E32)	p.2255,M>I	rs200325719	0.001	heterozygous	possibly related	Predict deleterious		
CD180	chr5:66479379	c.1292C>T(E3)	p.431,R>H	rs200803485	0	heterozygous	possibly related	Predict deleterious		
BIRC3	chr11:102201880	c.1232G>A(E6)	p.411,R>K	rs201361356	0.001	heterozygous	possibly related	Predict deleterious		
	chr11:102195289	c.49G>A(E2)	p.17,A>T	rs186307109	0.001	heterozygous	possibly related	Predict deleterious		
SYNPO2	chr4:119953186	c.3256T>C(E4)	p.1086,W>R	rs78841086	0.001	heterozygous	possibly related	Predict deleterious		
SPSB2	chr12:6980442	c.706G>A(E3)	p.236,R>C	rs61733180		heterozygous	possibly related	Predict deleterious		
OTOL1	chr3:161214909	c.314G>T(E1)	p.105,C>F	rs538130088	0	heterozygous	possibly related	Predict deleterious		
NMS	chr2:101089991	c.173G>A(E3)	p.58,R>H	rs201102943	0	heterozygous	possibly related	Predict deleterious		
FAT1	chr4:187540683	c.7057A>C(E10)	p.2353,S>A	rs146085516	0.005	heterozygous	possibly related	Predict deleterious		
FNDC1	chr6:159653498	c.1954C>T(E11)	p.652,R>C	rs552768977	0	heterozygous	possibly related	Predict deleterious		
AZU1	chr19:828263	c.92G>A(E2)	p.31,R>Q	rs144130932	0.001	heterozygous	possibly related	Predict deleterious		
TPCN1	chr12:113730855	c.2446C>G(E27)	p.816,L>V			heterozygous	possibly related	Predict deleterious		
CETN1	chr18:580853	c.445G>A(E1)	p.149,A>T	rs114933134	0.001	heterozygous	possibly related	Predict deleterious		
SPATA31A1	chr9:39360949	c.3187G>A(E4)	p.1063,A>T	rs62550833		homozygous	possibly related	Predict deleterious		
PARD3	chr10:34620267	c.2611G>A(E19)	p.871,P>S			heterozygous	possibly related	Predict deleterious		
								Predict		

MFSD6L	chr17:8701244	c.1195C>T(E1)	p.399,G>S	rs138080986	0.003	heterozygous	unkown	harmless		
ADC	chr1:33563692	c.941C>T(E10)	p.314,T>I			heterozygous	possibly related	Predict deleterious		
DENND3	chr8:142161820-142161836	c.718(E7) -c.734(E7):missing CTGCA<7>CACCC	missing frame shift			heterozygous	Relatively high	cause a significant change in the protein sequence		
SCGB3A1	chr5:180017690-180017690	c.201(E2):missing G	p.68,V>Xfs1	rs200868132	0.002	heterozygous	Relatively high	cause a significant change in the protein sequence		
MOSPD2	chrX:14937863	c.1300C>G(E14)	p.434,L>V	rs149352938		heterozygous	possibly related	Predict deleterious		
SEMA4B	chr15:90770839	c.1739C>T(E14)	p.580,A>V	rs143185707	0.004	heterozygous	possibly related	Predict deleterious		
C2orf15	chr2:99767073	c.154G>A(E4)	p.52,D>N	rs139891421	0.006	heterozygous	possibly related	Predict deleterious		
SLC26A9	chr1:205892250	c.1733C>T(E16)	p.578,R>K	rs185991098	0.001	heterozygous	possibly related	Predict deleterious		
LYZL4	chr3:42448377	c.253C>T(E3)	p.85,G>S	rs201569061	0.001	heterozygous	possibly related	Predict deleterious		
ZDHHC12	chr9:131483741	c.523G>A(E5)	p.175,R>W	rs141785453	0.005	heterozygous	possibly related	Predict deleterious		
AMIGO2	chr12:47471555	c.1231C>G(E3)	p.411,V>L	rs77621437	0.004	heterozygous	possibly related	Predict deleterious		
ANKRD27	chr19:33119653	c.1312C>T(E14)	p.438,D>N	rs145753923	0.005	heterozygous	possibly related	Predict deleterious		
SLIT3	chr5:168149292	c.2452C>T(E23)	p.818,A>T	rs202238818	0	heterozygous	possibly related	Predict deleterious		
SNAPC3	chr9:15422944	c.67G>A(E1)	p.23,G>S			heterozygous	possibly related	Predict deleterious		
								Predict		

FAM35A	chr10:88911427	c.316A>C(E3)	p.106,K>Q	rs138542761	0.008	heterozygous	possibly related	deleterious		
ZFP30	chr19:38126765	c.677C>A(E6)	p.226,G>V			heterozygous	possibly related	Predict deleterious		
CDC169-SOHLH2	chr13:36827969	c.139G>A(E4)	p.47,R>C	rs79770535	0.003	heterozygous	possibly related	Predict deleterious		
GZMA	chr5:54404053	c.458G>T(E4)	p.153,W>L	rs2270627	0.01	heterozygous	possibly related	Predict deleterious		
CCL3L3	chr17:34522695	c.272A>G(E3)	p.91,L>P	rs201343647		heterozygous	possibly related	Predict deleterious		
ACTR5	chr20:37400374	c.1739C>T(E9)	p.580,P>L	rs3752289	0.002	heterozygous	possibly related	Predict deleterious		
ATP5S	chr14:50789476	c.400G>A(E3)	p.134,D>N	rs17122388	0.01	heterozygous	possibly related	Predict deleterious		
DNAJC17	chr15:41099621	c.24T>G(E1)	p.8,L>F			heterozygous	possibly related	Predict deleterious		
LOC100996575	chr1:144619903	c.863T>A(E8)	p.288,V>E	rs71527379		homozygous	unkown	unkown		
HELZ	chr17:65105513	c.4208T>C(E29)	p.1403,Q>R	rs117052455	0.004	heterozygous	possibly related	Predict deleterious		
MTRNR2L2	chr5:79945871	c.35G>A(E1)	p.12,S>L	rs10942928		heterozygous	possibly related	Predict deleterious		
MTRNR2L8	chr11:10529739	c.35G>A(E1)	p.12,S>L	rs6484338		homozygous	possibly related	Predict deleterious		
C9orf43	chr9:116191187	c.1115C>T(E13)	p.372,T>M	rs143283507	0.009	heterozygous	possibly related	Predict deleterious		
	chr9:116188007-116188022	c.1027(E11) to IVS11+12: missing TATGG<6>GAATA	deletion shift			heterozygous	Relatively high	cause a significant change in the protein sequence		
FTSJD1	chr16:71319715	c.109C>T(E3)	p.37,G>S	rs192739719	0.004	heterozygous	possibly related	Predict deleterious		
C12orf50	chr12:88390214	c.418T>C(E6)	p.140,M>V	rs189756935	0.001	heterozygous	possibly related	Predict deleterious		

CCT4	chr2:62107466	c.244T>C(E3)	p.82,I>V	rs2272428	0.001	heterozygous	possibly related	Predict deleterious		
MUCL1	chr12:55250549	IVS2-5T>G	splice site	rs10783685		heterozygous	possibly related	mRNA splicing		
TNKS1BP1	chr11:57087740	c.541G>A(E3)	p.181,R>C			heterozygous	possibly related	Predict deleterious		
STEAP1B	chr7:22478220	c.917A>G(E5)	p.306,I>T	rs150559737	0.002	heterozygous	possibly related	Predict deleterious		
DONSON	chr21:34958445	c.445T>C(E3)	p.149,K>E			heterozygous	possibly related	Predict deleterious		
SLAMF8	chr1:159802845	c.547G>A(E3)	p.183,G>R	rs34249690	0.004	heterozygous	possibly related	Predict deleterious		
TM2D3	chr15:102182738	c.610C>T(E5)	p.204,V>I			heterozygous	possibly related	Predict deleterious		
FAM190B	chr10:86273280	c.2486C>T(E11)	p.829,T>M	rs61747212	0.008	heterozygous	possibly related	Predict deleterious		
AKR1E2	chr10:4875664	IVS3+6G>A	splice site	rs41289263	0.041	heterozygous	possibly related	mRNA splicing		
LHX9	chr1:197896778	c.764C>T(E5)	p.255,S>L			heterozygous	possibly related	Predict deleterious		
TCF20	chr22:42608151	c.3161G>C(E1)	p.1054,P>R			heterozygous	possibly related	Predict deleterious		
PRR14L	chr22:32109810	c.4015C>T(E4)	p.1339,E>K	rs567680239	0.002	heterozygous	possibly related	Predict deleterious		
PFKFB1	chrX:54989788	c.125G>C(E2)	p.42,P>R			heterozygous	possibly related	Predict deleterious		
CATSPERG	chr19:38851364	IVS15-7C>A	splice site	rs2286477	0.011	heterozygous	possibly related	mRNA splicing		
ENPP2	chr8:120608106	c.1109G>A(E12)	p.370,A>V	rs142695308	0.002	heterozygous	possibly related	Predict deleterious		
FAM22G	chr9:99698737	c.873G>A(E4)	p.291,M>I			heterozygous	possibly related	Predict deleterious		

TDRD3	chr13:61068657	c.806A>G(E8)	p.269,K>R	rs201585030	0	heterozygous	possibly related	Predict deleterious		
	chr13:61057936	c.523A>G(E6)	p.175,I>V	rs201745470	0	heterozygous	possibly related	Predict deleterious		
KCNMB3	chr3:178961073	IVS3-1C>G	splice site	rs191394366	0.001	heterozygous	Relatively high	mutation is at the classical shear site		
MYH7B	chr20:33570345	c.737C>T(E10)	p.246,P>L	rs149008892	0.002	heterozygous	possibly related	Predict deleterious		
PDE4DIP	chr1:144852390	c.7053C>T(E44)	p.2351,W>X(12)	rs61804988		heterozygous	Relatively high	nonsense mutation		
	chr1:144886092	IVS26+5C>T	splice site	rs1343472		heterozygous	possibly related	mRNA splicing		
BIN3	chr8:22481490	c.553G>A(E8)	p.185,R>C	rs11550511	0	heterozygous	possibly related	Predict deleterious		
OR8B2	chr11:124253123	c.117C>G(E1)	p.39,M>I	rs202110730	0.001	heterozygous	possibly related	Predict deleterious		
CEP350	chr1:180010896	c.4321A>G(E19)	p.1441,T>A	rs549806890	0.001	heterozygous	possibly related	Predict deleterious		
C12orf70	chr12:27627819	c.335A>G(E4)	p.112,Q>R			heterozygous	possibly related	Predict deleterious		
CCR9	chr3:45943130	c.814A>G(E4)	p.272,M>V	rs12721497	0.008	heterozygous	possibly related	Predict deleterious	19525985	
OR1D5	chr17:2966309	c.593G>A(E1)	p.198,A>V	rs148824825		heterozygous	possibly related	Predict deleterious		
	chr17:2966828	c.74C>T(E1)	p.25,R>Q	rs531952330	0.028	homozygous	possibly related	Predict deleterious		
FAM86B2	chr8:12291593	c.127C>A(E2)	p.43,D>Y	rs146321506		heterozygous	possibly related	Predict deleterious		
POLI	chr18:51810288	IVS6-4A>G	splice site	rs56194043	0	heterozygous	possibly related	mRNA splicing		
ABRA	chr8:107773278	c.1133G>A(E2)	p.378,T>M	rs200631708	0	heterozygous	possibly related	Predict deleterious		

RSPH3	chr6:159404706	c.906T>C(E4)	p.302,I>M			heterozygous	possibly related	Predict deleterious		
OR6C2	chr12:55846890	c.893C>T(E1)	p.298,A>V	rs74092333	0.036	heterozygous	possibly related	Predict deleterious		
GBF1	chr10:104117875	c.719T>C(E9)	p.240,M>T	rs143872476	0.007	heterozygous	possibly related	Predict deleterious		
CD48	chr1:160650919-160650919	c.725(E3):missing C	p.242,G>Vfs57	rs142100077	0.043	heterozygous	Relatively high			
SPDYE5	chr7:75130762	c.712C>T(E5)	p.238,R>C	rs587703560	0	heterozygous	possibly related	Predict deleterious		
SPATA31C1	chr9:90536994	c.2172A>C(E4)	p.724,Q>H	rs559309371	0.001	heterozygous	possibly related	Predict deleterious		
LUZP1	chr1:23417754	c.3001C>G(E3)	p.1001,E>Q	rs183227574	0	heterozygous	possibly related	Predict deleterious		
SPDYE4	chr17:8656662	c.631C>T(E5)	p.211,V>I	rs184746576	0.002	heterozygous	possibly related	Predict deleterious		
KRTAP24-1	chr21:31654638	c.613C>T(E1)	p.205,V>M	rs78287602	0.017	heterozygous	possibly related	Predict deleterious		
ADCY2	chr5:7766894	c.2189G>A(E17)	p.730,R>H	rs184691479		heterozygous	possibly related	Predict deleterious		
HRNR	chr1:152187935	c.6170C>T(E3)	p.2057,R>Q	rs61814938		heterozygous	possibly related	Predict deleterious		
	chr1:152191809	c.2296C>T(E3)	p.766,G>S	rs142594836	0.005	heterozygous	possibly related	Predict deleterious		
ADCY3	chr2:25050847	c.2356C>T(E13)	p.786,A>T	rs78678013		heterozygous	possibly related	Predict deleterious		
RNF111	chr15:59323359	c.338A>G(E2)	p.113,Q>R			heterozygous	possibly related	Predict deleterious		
ZBED4	chr22:50277352	c.42T>G(E2)	p.14,D>E	rs80100118	0.006	heterozygous	possibly related	Predict deleterious		
								Predict		

ABP1	chr7:150554255	c.697G>A(E2)	p.233,V>M	rs201353384	0.001	heterozygous	possibly related	deleterious		
SAMD14	chr17:48190340	c.1171C>T(E10)	p.391,E>K	rs201142482	0.001	heterozygous	possibly related	Predict deleterious		
CREB5	chr7:28610022	c.310G>A(E5)	p.104,G>R			heterozygous	possibly related	Predict deleterious		
SMC5	chr9:72967219	c.3278G>A(E25)	p.1093,R>H	rs201772265	0	heterozygous	possibly related	Predict deleterious		
KLK3	chr19:51361503	c.425C>T(E3)	p.142,P>L			heterozygous	possibly related	Predict deleterious		
SAMD12	chr8:119593108	c.38C>T(E2)	p.13,R>Q	rs145316106		heterozygous	possibly related	Predict deleterious		
NES	chr1:156640385	c.3595C>G(E4)	p.1199,A>P	rs78303930	0.005	heterozygous	possibly related	Predict deleterious		
AVIL	chr12:58201191	c.1414C>G(E12)	p.472,V>L	rs140328274	0	heterozygous	possibly related	Predict deleterious		
OR9I1	chr11:57886435	c.482G>T(E1)	p.161,T>N	rs76688946	0.007	heterozygous	possibly related	Predict deleterious		
CHD9	chr16:53191365	c.1364A>C(E2)	p.455,Q>P	rs545880354	0.001	heterozygous	possibly related	Predict deleterious		
CCDC121	chr2:27850162	c.991T>G(E2)	p.331,K>Q			heterozygous	possibly related	Predict deleterious		
VCAM1	chr1:101198118	c.1670C>T(E7)	p.557,P>L			heterozygous	possibly related	Predict deleterious		
NAALADL1	chr11:64822188	c.626C>T(E5)	p.209,G>E	rs193248866	0	heterozygous	possibly related	Predict deleterious		
UPP2	chr2:158958551-158958552	IVS2-1 to c.148(E3):insert A	insert frame shift	rs67998287		homozygous	Relatively high	mutation in initiation codon, Affect protein coding initiation site		
TAS2R10	chr12:10978607	c.262A>G(E1)	p.88,W>R	rs117936881	0.008	heterozygous	possibly related	Predict deleterious		
C1orf27	chr1:186355175	c.290C>T(E4)	p.97,T>I			heterozygous	possibly related	Predict deleterious		

CASQ1	chr1:160160543	c.2T>A(E1)	p.1,M>K	rs189183669	0.001	heterozygous	Relatively high	mutation in initiation codon，Affect protein coding initiation site		
JMJD1C	chr10:64974548	c.722A>G(E5)	p.241,M>T	rs151186255	0.009	heterozygous	possibly related	Predict deleterious		
CNTN4	chr3:3072577	c.1701A>C(E15)	p.567,Q>H	rs181710158	0.002	heterozygous	possibly related	Predict deleterious		
SPEF2	chr5:35654781	c.931C>T(E7)	p.311,R>W	rs574492016	0	heterozygous	possibly related	Predict deleterious		
FAM120C	chrX:54117740	IVS11+5C>A	splice site	rs2495797		homozygous	possibly related	mRNA splice		
UFL1	chr6:96974210	c.364C>T(E5)	p.122,R>W	rs146188768	0.001	heterozygous	possibly related	Predict deleterious		
TRPM2	chr21:45833864	c.3053C>T(E20)	p.1018,P>L	rs145947009	0.007	heterozygous	possibly related	Predict deleterious		
KIDINS220	chr2:8890355	c.3301C>T(E24)	p.1101,V>M	rs561743411	0	heterozygous	possibly related	Predict deleterious		
KRTAP27-1	chr21:31709703	c.284C>T(E1)	p.95,C>Y	rs117534873	0.016	heterozygous	possibly related	Predict deleterious		
CES5A	chr16:55899922	c.658C>T(E5)	p.220,V>M	rs145397395	0.001	heterozygous	possibly related	Predict deleterious		
ATP6V1H	chr8:54745584	c.176G>A(E3)	p.59,P>L			heterozygous	possibly related	Predict deleterious		
TRPM5	chr11:2429014	c.2911T>G(E19)	p.971,M>L	rs199966281	0	heterozygous	possibly related	Predict deleterious		
ANKRD36B	chr2:98128313	c.3008G>A(E39)	p.1003,T>M	rs202131502		heterozygous	possibly related	Predict deleterious		
	chr2:98128421	c.2900T>C(E39)	p.967,K>R	rs200637231		heterozygous	possibly related	Predict deleterious		
	chr2:98128515	c.2806C>T(E39)	p.936,V>I	rs202184678		heterozygous	possibly related	Predict deleterious		
SLC43A1	chr11:57261526	c.811C>T(E8)	p.271,G>S			heterozygous	possibly related	Predict deleterious		

ALS2CR11	chr2:202356749	c.4315T>C(E15)	p.1439,K>E	rs75689388	0.042	heterozygous	possibly related	Predict deleterious		
	chr2:202352433	c.5365G>A(E16)	p.1789,R>C	rs117420067	0.01	heterozygous	possibly related	Predict deleterious		
NBPF1	chr1:16903912	c.1804T>A(E18)	p.602,N>Y	rs61772344		homozygous	unkown	unkown		
	chr1:16901668	c.2176T>C(E20)	p.726,K>E	rs3901679		heterozygous	possibly related	Predict deleterious		
	chr1:16902884	c.1997T>C(E19)	p.666,N>S	rs74630591		heterozygous	possibly related	Predict deleterious		
	chr1:16905718	c.1771A>C(E17)	p.591,C>G	rs3738661		heterozygous	possibly related	Predict deleterious		
C7orf72	chr7:50173835	c.860T>C(E4)	p.287,I>T	rs139478246	0.002	heterozygous	possibly related	Predict deleterious		
L1TD1	chr1:62675798	c.1352T>C(E5)	p.451,L>S	rs542429784		heterozygous	possibly related	Predict deleterious		
SH2D4A	chr8:19221675	c.799G>A(E7)	p.267,G>R			heterozygous	possibly related	Predict deleterious		
ACOT9	chrX:23726013-23726015	c.684(E9) to c.682(E9):missing CTC	missing non-frameshift			heterozygous	unkown	unkown		
TMF1	chr3:69097297	c.559T>C(E2)	p.187,M>V	rs76863529	0.001	heterozygous	possibly related	Predict deleterious		
TMEM232	chr5:109954273	IVS7-10T>C	splice site	rs139336689	0.004	heterozygous	possibly related	mRNA splice		
PRDX2	chr19:12911709	c.278C>T(E3)	p.93,R>K	rs34577345	0.003	heterozygous	possibly related	Predict deleterious		
LRRC8C	chr1:90178308	c.179A>G(E3)	p.60,Q>R	rs201902728	0	heterozygous	possibly related	Predict deleterious		
E2F7	chr12:77423619	c.1876T>C(E10)	p.626,M>V	rs3829295	0.008	heterozygous	possibly related	Predict deleterious		
SEC24A	chr5:133997171	c.460C>T(E2)	p.154,R>C	rs79561592	0.007	heterozygous	possibly related	Predict deleterious		

IMPA1	chr8:82592967	c.292G>A(E4)	p.98,P>S	rs61755740	0.001	heterozygous	possibly related	Predict deleterious		
XPO7	chr8:21842329	c.1450A>G(E12)	p.484,M>V	rs201923651	0	heterozygous	possibly related	Predict deleterious		
KRTAP19-7	chr21:31933451	c.158C>T(E1)	p.53,C>Y	rs148576261	0.008	heterozygous	possibly related	Predict deleterious		
NREP	chr5:111312435	c.2A>G(E1)	p.1,M>T	rs562091442	0	heterozygous	Relatively high	mutation in initiation codon，Affect protein coding initiation site		
MYO10	chr5:16690033	IVS27-5A>C	splice site	rs2303704	0.044	heterozygous	possibly related	mRNA splice		
APEH	chr3:49719799	c.1622C>T(E18)	p.541,T>M	rs3816877	0.007	heterozygous	possibly related	Predict deleterious		
FBXO39	chr17:6683848	c.661T>A(E2)	p.221,S>T	rs4143218	0.009	heterozygous	possibly related	Predict deleterious		
DNAJB8	chr3:128181970	c.119T>C(E3)	p.40,E>G	rs201512803	0.001	heterozygous	possibly related	Predict deleterious		
MUC4	chr3:195506197	c.12254T>C(E2)	p.4085,D>G	rs148307810	0.007	heterozygous	possibly related	Predict deleterious		
	chr3:195515861	c.2590C>A(E2)	p.864,A>S	rs376032160	0	heterozygous	possibly related	Predict deleterious		
FAM208B	chr10:5804509	c.7189C>T(E20)	p.2397,P>S	rs75004071	0.002	heterozygous	unkown	Predict harmless		
	chr10:5789041-5789045	c.3657(E15) to c.3661(E15): missing TTCAC	p.1220,S>Xfs1			heterozygous	Relatively high	cause a significant change in the protein sequence		
DNHD1	chr11:6524126	c.890A>G(E4)	p.297,Y>C	rs78466420	0.005	heterozygous	possibly related	Predict deleterious		
FDPS	chr1:155279968	c.311T>C(E3)	p.104,I>T			heterozygous	possibly related	Predict deleterious		
								Predict		

TMEM225	chr11:123753958	c.565C>T(E4)	p.189,E>K	rs35551906	0.041	heterozygous	possibly related	deleterious		
SYT10	chr12:33579218	c.364G>C(E2)	p.122,P>A	rs185570590	0.002	heterozygous	possibly related	Predict deleterious		
PALM2-AKAP2	chr9:112899278	c.1454T>C(E8)	p.485,L>P			heterozygous	possibly related	Predict deleterious		
PHACTR2	chr6:144070193	c.286C>G(E3)	p.96,P>A			heterozygous	possibly related	Predict deleterious		
OR2T35	chr1:248801610	c.950G>A(E1)	p.317,A>V	rs150878651	0.003	heterozygous	possibly related	Predict deleterious		
	chr1:248801611	c.949C>T(E1)	p.317,A>T	rs143981271		heterozygous	possibly related	Predict deleterious		
APOBR	chr16:28508245	c.1883C>G(E2)	p.628,T>R	rs13306186	0.003	heterozygous	possibly related	Predict deleterious		
P2RY4	chrX:69478736	c.739T>C(E1)	p.247,I>V	rs56217451		heterozygous	possibly related	Predict deleterious		
C20orf26	chr20:20243759-20243760	c.2488(E21) to c.2489(E21): insert C	p.830,S>Sfs16			heterozygous	Relatively high	cause a significant change in the protein sequence		
	chr20:20054946	c.295C>T(E3)	p.99,P>S			heterozygous	possibly related	Predict deleterious		
SLC38A10	chr17:79254485	c.550G>A(E6)	p.184,R>W	rs200753630	0.004	heterozygous	possibly related	Predict deleterious		
ANKRD30B	chr18:14852142	c.3842T>G(E36)	p.1281,M>R	rs188634046	0.002	heterozygous	possibly related	Predict deleterious		
LRRK1	chr15:101606215	c.5573G>A(E32)	p.1858,S>N	rs34874770	0.001	heterozygous	possibly related	Predict deleterious		
INTU	chr4:128554293	c.104T>C(E1)	p.35,V>A	rs117754102	0.007	heterozygous	possibly related	Predict deleterious		
GPR50	chrX:150349558-150349569	c.1503(E2) to c.1514(E2): missing CACCACTGGCCA	missing non-frameshift	rs68058591		heterozygous	possibly related	Predict deleterious		

MARC1	chr1:220978568	c.839C>T(E6)	p.280,P>L	rs139463105	0.001	heterozygous	possibly related	Predict deleterious		
KRTAP26-1	chr21:31691792	c.562G>T(E1)	p.188,P>T	rs12483584	0.016	heterozygous	possibly related	Predict deleterious		
KIAA1467	chr12:13215794	c.737C>T(E5)	p.246,T>I			heterozygous	possibly related	Predict deleterious		
TMEM204	chr16:1584438	c.162G>C(E2)	p.54,R>S	rs186729006	0.008	homozygous	possibly related	Predict deleterious		
RPS27A	chr2:55461964	IVS4-3C>A	splice site	rs118100576	0.007	heterozygous	possibly related	mRNA splice		
MTUS1	chr8:17570729	c.2617T>G(E6)	p.873,N>H	rs187103704	0.002	heterozygous	Relatively high	report of diseases	16650523	
IL1A	chr2:113535591	c.588T>G(E6)	p.196,Q>H			heterozygous	possibly related	Predict deleterious		
ZNF302	chr19:35175607	c.665T>C(E5)	p.222,I>T	rs374913967	0.001	heterozygous	possibly related	Predict deleterious		
PGLYRP4	chr1:153314126	c.602C>T(E6)	p.201,R>Q	rs148195147	0.003	heterozygous	possibly related	Predict deleterious		
UNC5B	chr10:73051568	c.1641C>G(E9)	p.547,I>M	rs150945696	0.005	heterozygous	possibly related	Predict deleterious		
NRCAM	chr7:107849964	c.976G>C(E9)	p.326,Q>E			heterozygous	possibly related	Predict deleterious		
AGAP5	chr10:75442543	c.406C>T(E5)	p.136,E>K	rs200625436		heterozygous	possibly related	Predict deleterious		
OR52D1	chr11:5510553	c.617C>T(E1)	p.206,A>V	rs142747961	0.007	heterozygous	possibly related	Predict deleterious		
ZXDA	chrX:57935224	c.1631C>T(E1)	p.544,R>H	rs201671903		heterozygous	possibly related	Predict deleterious		
RPS6KB2	chr11:67201730	c.1031C>T(E12)	p.344,P>L	rs200448294		heterozygous	possibly related	Predict deleterious		
KNTC1	chr12:123082465	c.4543C>G(E44)	p.1515,L>V	rs188333792	0.001	heterozygous	possibly related	Predict deleterious		

DAK	chr11:61110874	c.926T>C(E11)	p.309,M>T			heterozygous	possibly related	Predict deleterious		
XPO5	chr6:43541257	c.187C>T(E2)	p.63,V>I	rs370390582	0.001	heterozygous	possibly related	Predict deleterious		
MYH15	chr3:108110718	c.5379C>G(E38)	p.1793,R>S	rs139977838	0	heterozygous	possibly related	Predict deleterious		
MICB	chr6:31477681	c.1147A>G(E6)	p.383,T>A			homozygous	possibly related	Predict deleterious		
	chr6:31473546	c.223A>G(E2)	p.75,N>D			homozygous	possibly related	Predict deleterious		
ZNF768	chr16:30536994	c.467A>G(E2)	p.156,L>P	rs143274508	0.004	heterozygous	possibly related	Predict deleterious		
KATNAL2	chr18:44585943	c.251C>G(E5)	p.84,T>S	rs142467674	0.001	homozygous	possibly related	Predict deleterious		
CWC22	chr2:180846612	c.319A>T(E5)	p.107,S>T			heterozygous	possibly related	Predict deleterious		
USP47	chr11:11964006	c.2234C>T(E19)	p.745,P>L	rs563749674	0	heterozygous	possibly related	Predict deleterious		
WDR27	chr6:170062481	c.563C>T(E6)	p.188,G>E	rs117912086	0.005	heterozygous	possibly related	Predict deleterious		
ZG16	chr16:29790930-29790935	c.160(E3) to c.165(E3): missing GTCCGA	missing non-frameshift	rs200571875	0.011	heterozygous	possibly related	Predict deleterious		
GANC	chr15:42631977	c.1954C>T(E17)	p.652,R>X(263)			heterozygous	Relatively high	Early termination of protein translation		
ZNF670	chr1:247201524	c.394A>G(E4)	p.132,C>R			heterozygous	possibly related	Predict deleterious		
RLF	chr1:40702974	c.2600C>A(E8)	p.867,P>H	rs78728589	0.009	heterozygous	possibly related	Predict deleterious		
	chr1:40704937	c.4563C>G(E8)	p.1521,I>M	rs75855852	0.009	heterozygous	possibly related	Predict deleterious		
								Predict		

RASSF3	chr12:65078611	c.156G>C(E2)	p.52,E>D	rs574359875	0	heterozygous	possibly related	deleterious		
ZNF23	chr16:71483462	c.466C>T(E6)	p.156,V>M	rs575375073	0	heterozygous	possibly related	Predict deleterious		
STAB2	chr12:104031859	c.775C>T(E8)	p.259,R>W	rs578254735	0	heterozygous	possibly related	Predict deleterious		
PDZRN4	chr12:41961613	c.1496G>A(E9)	p.499,R>K	rs193168225	0.003	heterozygous	possibly related	Predict deleterious		
TMPRSS9	chr19:2416601	c.1709C>T(E11)	p.570,P>L	rs146529608	0	heterozygous	possibly related	Predict deleterious		
ZNF667	chr19:56952821	c.1543C>T(E5)	p.515,A>T	rs201561717	0.001	heterozygous	possibly related	Predict deleterious		
ATRN	chr20:3541478	c.1025G>A(E8)	p.342,R>Q	rs200822955		heterozygous	possibly related	Predict deleterious		
NBPF10	chr1:145330829	c.4709A>G(E36)	p.1570,H>R	rs376830820		heterozygous	possibly related	Predict deleterious		
	chr1:145360584	c.9209G>A(E74)	p.3070,G>E	rs368319396		homozygous	possibly related	Predict deleterious		
	chr1:145327548	c.4105A>G(E32)	p.1369,N>D	rs202019968		heterozygous	possibly related	Predict deleterious		
NBPF16	chr1:148754942	c.1598A>T(E14)	p.533,Q>L	rs201022397		heterozygous	possibly related	Predict deleterious		
	chr1:148754858	c.1514T>C(E14)	p.505,V>A	rs199921772		heterozygous	possibly related	Predict deleterious		
ABCA10	chr17:67187340	c.1988A>G(E18)	p.663,L>S	rs138284687	0.003	heterozygous	possibly related	Predict deleterious		
TTC14	chr3:180327501	c.1484C>T(E12)	p.495,S>F	rs118079716	0.007	heterozygous	possibly related	Predict deleterious		
NBPF14	chr1:148010911	c.1711G>A(E14)	p.571,R>C	rs61810210		heterozygous	possibly related	Predict deleterious		
TBC1D3	chr17:36339597	c.1060G>T(E13)	p.354,Q>K	rs373252714		heterozygous	possibly related	Predict deleterious		
								Predict		

MYOM2	chr8:2017449	c.706G>A(E7)	p.236,G>R	rs149104727		heterozygous	possibly related	deleterious		
COL6A6	chr3:130305358	c.3979G>A(E10)	p.1327,A>T	rs138732186	0.001	heterozygous	possibly related	Predict deleterious		
GALNT10	chr5:153765904	c.970G>A(E7)	p.324,V>M			heterozygous	possibly related	Predict deleterious		
SORBS3	chr8:22426701	c.320C>T(E6)	p.107,P>L	rs77246845	0.004	heterozygous	possibly related	Predict deleterious		
UEVLD	chr11:18553921	c.1362T>A(E12)	p.454,K>N	rs201635931	0.002	heterozygous	possibly related	Predict deleterious		
CYP2A13	chr19:41600311	c.1135A>C(E7)	p.379,K>Q	rs115698903	0.017	heterozygous	possibly related	Predict deleterious		
UNC79	chr14:94088884	c.4774A>G(E30)	p.1592,T>A			heterozygous	possibly related	Predict deleterious		
PLEKHG4	chr16:67319381	c.2384G>A(E15)	p.795,R>H	rs199603402		heterozygous	possibly related	Predict deleterious		
NHLRC2	chr10:115618430	c.322T>C(E2)	p.108,S>P	rs201956750	0.001	heterozygous	possibly related	Predict deleterious		
KANSL1L	chr2:210887734	c.2903T>C(E15)	p.968,D>G	rs117674897	0.004	heterozygous	possibly related	Predict deleterious		
ZNF556	chr19:2877426	c.470C>T(E4)	p.157,T>I	rs34849844	0.007	heterozygous	possibly related	Predict deleterious		
RABGAP1	chr9:125761051	IVS10+6G>A	splice site	rs605546		heterozygous	possibly related	mRNA splice		
KIAA1217	chr10:24813277	c.2242G>A(E14)	p.748,G>S	rs201731110	0.001	heterozygous	possibly related	Predict deleterious		
GRIP2	chr3:14583420	c.169G>A(E1)	p.57,P>S	rs139005453	0.002	heterozygous	possibly related	Predict deleterious		
UNC80	chr2:210836943	c.8077C>A(E54)	p.2693,P>T	rs577801702	0	heterozygous	possibly related	Predict deleterious		
SRGAP3	chr3:9055458	c.1810T>C(E16)	p.604,I>V	rs2271207	0.005	heterozygous	possibly related	Predict deleterious		
								Predict		

ALG10B	chr12:38714237	c.644A>G(E3)	p.215,K>R	rs183263763	0.003	heterozygous	possibly related	deleterious		
DECR1	chr8:91057221	c.883G>A(E8)	p.295,A>T	rs201405525	0.001	heterozygous	possibly related	Predict deleterious		
ZNF337	chr20:25655955	c.1969C>G(E5)	p.657,V>L	rs146126391		heterozygous	possibly related	Predict deleterious		
ZNF479	chr7:57187745	c.1377A>T(E5)	p.459,H>Q	rs375939463		heterozygous	possibly related	Predict deleterious		
CXorf59	chrX:36162684-36162685	c.1267(E11) to c.1268(E11): insert TG	p.423,L>Lfs2	rs372871921		heterozygous	Relatively high	cause a significant change in the protein sequence		
FBXL21	chr5:135276202-135276205	c.514(E7) to c.517(E7): missing GTTG insert C	complex non-frameshift	rs1160982,rs568134250		heterozygous	unkown	unkown		
ZNF285	chr19:44892034	c.373C>G(E4)	p.125,V>L	rs201529972	0.001	heterozygous	possibly related	Predict deleterious		
TMCO6	chr5:140021612	c.472C>A(E4)	p.158,L>I	rs76244220	0.002	heterozygous	possibly related	Predict deleterious		
ZNF33A	chr10:38343647	c.595A>G(E5)	p.199,T>A			heterozygous	possibly related	Predict deleterious		
MRPL1	chr4:78784022	c.20G>C(E1)	p.7,C>S			heterozygous	possibly related	Predict deleterious		
ARHGEF3	chr3:56763357	c.1618G>A(E13)	p.540,R>C	rs115250058	0.002	heterozygous	possibly related	Predict deleterious		
TTC31	chr2:74717206	c.184C>T(E3)	p.62,R>W	rs202055795	0.002	heterozygous	possibly related	Predict deleterious		
ZDHHC1	chr16:67434917	c.371C>T(E4)	p.124,R>Q	rs34229857	0.01	heterozygous	possibly related	Predict deleterious		
COPB2	chr3:139097997	c.247C>T(E4)	p.83,V>M	rs79043251	0.002	heterozygous	possibly related	Predict deleterious		
DDI1	chr11:103908689	c.1139C>T(E1)	p.380,S>L	rs75116260	0.004	heterozygous	possibly related	Predict deleterious		
								Predict		

RTN3	chr11:63525703	c.485C>T(E4)	p.162,T>I			heterozygous	possibly related	deleterious		
KRT33B	chr17:39521751	c.643C>G(E4)	p.215,A>P	rs143499346	0.008	heterozygous	possibly related	Predict deleterious		
OR7E24	chr19:9362138	c.419G>A(E1)	p.140,R>Q	rs180840288	0.002	heterozygous	possibly related	Predict deleterious		
ZNF880	chr19:52887146-52887146	c.313(E4): missing A	p.105,K>Kfs6	rs398101268		heterozygous	Relatively high	cause a significant change in the protein sequence		
LRRC18	chr10:50121457	c.744C>A(E1)	p.248,K>N	rs186297793	0	heterozygous	possibly related	Predict deleterious		
TLL2	chr10:98180756	c.880C>T(E7)	p.294,D>N	rs151093714		heterozygous	possibly related	Predict deleterious		
LOC401052	chr3:10050110	c.55C>T(E3)	p.19,A>T	rs185135517	0.007	heterozygous	possibly related	Predict deleterious		
FCGBP	chr19:40434108	c.161C>T(E2)	p.54,R>H			heterozygous	possibly related	Predict deleterious		
CDC42BPA	chr1:227327354	c.1313T>C(E10)	p.438,N>S			heterozygous	possibly related	Predict deleterious		
HSPA12B	chr20:3729935	c.904G>A(E9)	p.302,V>I			heterozygous	possibly related	Predict deleterious		
WDR66	chr12:122359397-122359398	c.186(E2) to c.187(E2):insert GAGGAGGAGGAGAAA	insert non-frameshift	rs142042908		heterozygous	unkown	Predict harmless		
TSGA10IP	chr11:65721129	c.1243G>A(E5)	p.415,V>M	rs147122262	0.003	heterozygous	possibly related	Predict deleterious		
KCNK2	chr1:215408418	c.1199G>A(E7)	p.400,S>N	rs76873069	0.001	heterozygous	possibly related	Predict deleterious		
CDC20B	chr5:54442491	c.320A>T(E3)	p.107,L>Q	rs148902887	0.001	heterozygous	possibly related	Predict deleterious		
PPFIBP2	chr11:7614412	c.329G>A(E4)	p.110,R>H			heterozygous	possibly related	Predict deleterious		

NXF2	chrX:101576806	IVS16+1G>A	splice site	rs60955190		heterozygous	Relatively high	mutation at the classic splice site		
MAP2K3	chr17:21217508	c.923C>T(E12)	p.308,A>V	rs186277389		heterozygous	possibly related	Predict deleterious		
TAOK1	chr17:27869597	c.2563G>A(E20)	p.855,A>T	rs34151057	0.003	heterozygous	possibly related	Predict deleterious		
MKI67	chr10:129903199	c.5825C>G(E12)	p.1942,W>S			heterozygous	possibly related	Predict deleterious		
	chr10:129914017	c.655T>G(E7)	p.219,K>Q	rs199752000	0.001	heterozygous	possibly related	Predict deleterious		
GUCY2F	chrX:108718478	c.688G>A(E2)	p.230,R>W	rs33973457		homozygous	possibly related	Predict deleterious		
	chrX:108696981	c.1140C>A(E4)	p.380,Q>H	rs2272925		homozygous	possibly related	Predict deleterious		
	chrX:108647653	c.2029C>G(E10)	p.677,V>L	rs35474112		homozygous	possibly related	Predict deleterious		
AKD1	chr6:109830547	c.4316T>A(E33)	p.1439,H>L	rs183899474	0.003	heterozygous	possibly related	Predict deleterious		
C17orf80	chr17:71238491	IVS4+1G>A	splice site	rs3764365	0.002	heterozygous	Relatively high	mutation at the classic splice site		
AHNAK2	chr14:105409808	c.11980T>A(E7)	p.3994,T>S	rs200747295	0.005	heterozygous	possibly related	Predict deleterious		
TMEM123	chr11:102272839	c.256C>A(E3)	p.86,V>F	rs11547915	0.02	heterozygous	possibly related	Predict deleterious		
KBTBD6	chr13:41706628	c.20G>A(E1)	p.7,A>V	rs200762614	0.003	heterozygous	possibly related	Predict deleterious		
ZNF226	chr19:44680896	c.1481A>C(E6)	p.494,Q>P			heterozygous	possibly related	Predict deleterious		
GOLGA6D	chr15:75586737	c.2003T>C(E18)	p.668,V>A	rs200428716		homozygous	possibly related	Predict deleterious		
HSPA1L	chr6:31779620	c.130C>T(E2)	p.44,V>M	rs189710538	0.001	heterozygous	possibly related	Predict deleterious		

CCDC169	chr13:36827969	c.439G>A(E6)	p.147,R>C	rs79770535	0.003	heterozygous	possibly related	Predict deleterious		
CCDC168	chr13:103386261	c.16786T>C(E4)	p.5596,I>V	rs139301837	0.001	heterozygous	possibly related	Predict deleterious		
ZNF619	chr3:40529139	c.1258G>A(E6)	p.420,A>T	rs202034818	0.001	heterozygous	possibly related	Predict deleterious		
OR2T4	chr1:248525576	c.694T>C(E1)	p.232,Y>H			heterozygous	possibly related	Predict deleterious		
FCHSD1	chr5:141024193	c.1589G>T(E16)	p.530,S>Y	rs2291110	0	heterozygous	possibly related	Predict deleterious		
SCYL1	chr11:65294315	c.664C>T(E5)	p.222,R>W			heterozygous	possibly related	Predict deleterious		
CD163L1	chr12:7519826	IVS18+6A>G	splice site	rs7974006		homozygous	possibly related	mRNA splice		
DOC2A	chr16:30018229	c.755C>T(E8)	p.252,R>H			heterozygous	possibly related	Predict deleterious		
ZNF233	chr19:44778796-44778799	c.1983(E5) to c.1986(E5):missing GTTG insert ATA	complex frameshift	rs59660444		heterozygous	Relatively high	frameshift mutation		
PTPRG	chr3:62263245	c.3656A>C(E26)	p.1219,E>A			heterozygous	possibly related	Predict deleterious		
MYH1	chr17:10406520	c.2737T>C(E23)	p.913,I>V			heterozygous	possibly related	Predict deleterious		
MAP2K5	chr15:68099048	c.1199G>A(E22)	p.400,R>Q			heterozygous	possibly related	Predict deleterious		
INTS5	chr11:62415882	c.1670C>G(E2)	p.557,C>S			heterozygous	possibly related	Predict deleterious		
CCDC141	chr2:179730607	c.2611G>A(E17)	p.871,Q>X(660)			heterozygous	Relatively high	nonsense mutation		
ACBD5	chr10:27512335	c.317C>T(E5)	p.106,R>H	rs77955449	0.002	heterozygous	possibly related	Predict deleterious		

MAGEC3	chrX:140985142	c.1598A>G(E7)	p.533,Y>C	rs199889482		heterozygous	possibly related	Predict deleterious		
KIAA0319L	chr1:35944780	c.700T>C(E4)	p.234,T>A	rs144951042	0.002	heterozygous	possibly related	Predict deleterious		

copy number variation(CNV):

<table>
<tr><th>CNV type: copy number</th><th>Chromosome name</th><th>initial position</th><th>termination position</th><th>CNV size</th><th>Number of genes in this region</th><th>Number of associated diseases</th><th>gene name</th><th>gene function or Associated disease phenotype</th></tr>
<tr><td rowspan="2">repetition 1</td><td rowspan="2">chr15</td><td rowspan="2">20170066</td><td rowspan="2">22842114</td><td rowspan="2">2672Kb</td><td rowspan="2">9</td><td rowspan="2">1</td><td>15q11-q13</td><td>Chromosome 15q11-q13 duplication syndrome</td></tr>
<tr><td>LOC642131; GOLGA6L1; TUBGCP5; LOC646057; OR4M2; OR4N4; LOC100996331; LOC100287399; GOLGA6L6</td><td></td></tr>
</table>
